# Unraveling the Tissue-Specific Gene Signatures of Gilthead Sea Bream (*Sparus aurata* L.) after Hyper- and Hypo-Osmotic Challenges

**DOI:** 10.1371/journal.pone.0148113

**Published:** 2016-02-01

**Authors:** Juan Antonio Martos-Sitcha, Juan Miguel Mancera, Josep Alvar Calduch-Giner, Manuel Yúfera, Gonzalo Martínez-Rodríguez, Jaume Pérez-Sánchez

**Affiliations:** 1 Instituto de Ciencias Marinas de Andalucía, Consejo Superior de Investigaciones Científicas (ICMAN-CSIC), E-11519, Puerto Real (Cádiz), Spain; 2 Department of Biology, Faculty of Marine and Environmental Sciences, Campus de Excelencia Internacional del Mar (CEI-MAR), University of Cádiz, E-11519, Puerto Real (Cádiz), Spain; 3 Nutrigenomics and Fish Growth Endocrinology Group, Institute of Aquaculture Torre de la Sal, Consejo Superior de Investigaciones Científicas (IATS-CSIC), Ribera de Cabanes, E-12595, Castellón, Spain; Chinese Academy of Fishery Sciences, CHINA

## Abstract

A custom microarray was used for the transcriptomic profiling of liver, gills and hypothalamus in response to hypo- (38‰ → 5‰) or hyper- (38‰ → 55‰) osmotic challenges (7 days after salinity transfer) in gilthead sea bream (*Sparus aurata*) juveniles. The total number of differentially expressed genes was 777. Among them, 341 and 310 were differentially expressed in liver after hypo- and hyper-osmotic challenges, respectively. The magnitude of changes was lower in gills and hypothalamus with around 131 and 160 responsive genes in at least one osmotic stress condition, respectively. Regardless of tissue, a number of genes were equally regulated in either hypo- and hyper-osmotic challenges: 127 out of 524 in liver, 11 out of 131 in gills and 19 out of 160 in hypothalamus. In liver and gills, functional analysis of differentially expressed genes recognized two major clusters of overlapping canonical pathways that were mostly related to “Energy Metabolism” and “Oxidative Stress”. The later cluster was represented in all the analyzed tissues, including the hypothalamus, where differentially expressed genes related to “Cell and tissue architecture” were also over-represented. Overall the response for “Energy Metabolism” was the up-regulation, whereas for oxidative stress-related genes the type of response was highly dependent of tissue. These results support common and different osmoregulatory responses in the three analyzed tissues, helping to load new allostatic conditions or even to return to basal levels after hypo- or hyper-osmotic challenges according to the different physiological role of each tissue.

## Introduction

Euryhaline teleosts can live in a wide range of environmental salinities, maintaining the osmolality of their internal fluids between certain values regardless of the external osmolality [1, 2]. The existence of different environmental osmotic conditions imposes a passive flux of water and ions between internal and external milieus that forces the specimens to face different osmoregulatory strategies. Accordingly, two different mechanisms of osmoregulatory processes can be considered: i) a hypoosmotic regulation in animals living in salinities above the isosmotic point (~12–15‰ salinity), as occurred in seawater (SW, ~36–38‰ salinity) adapted teleosts; and ii) a hyperosmotic regulation in lower salinities, as is arisen in freshwater (FW, ~0‰ salinity) adapted fish [3–5]. Both processes are driven, among other osmoregulatory tissues, by the gills, a multipurpose organ that has evolved for aquatic gas exchange, but also for dominant roles in osmotic and ionic regulation, acid-base regulation and excretion of nitrogenous wastes [6]. At the metabolic level, the maintenance of an internal ionic composition distinct from that of the environment is a highly consuming energy process at the local tissue level [7, 8]. At the same time, the liver is considered a high sensitive and responsive tissue to changes in energy demand in response, among others, to osmotic, thermal and nutritional stressors [7, 9, 10]. This metabolic response must be controlled by the central nervous system, where hypothalamus is considered a key upstream element of the osmoregulatory mediated response of modern fish (see [2, 11] for review). However, the molecular phenotyping of osmotic-stress response remains still far to be resolved, especially when it is considered in a tissue-integrated manner.

After almost 20 years of development, DNA microarray technology is mature, and along the years of its existence it has successfully evolved for the simultaneous profiling of a huge number of genes to recognize complex gene expression patterns [12–14]. Particularly, in gilthead sea bream (*Sparus aurata* L.), different microarrays have been used to assess the transcriptional-mediated changes that occurs during ontogeny [15], skin and scales regeneration [16] and stress-responses induced by cortisol administration [17, 18], confinement exposure [19] or parasite and nutritional challenges as a result of changes in diet composition and/or ration size [20–22].

The aim of the present study was to describe new aspects related to osmoregulatory processes not previously addressed by classical methodologies, analyzing the effects of hyper- and hypo-osmoregulatory challenges in different tissues of gilthead sea bream (*S*. *aurata*) using a custom oligo-microarray. This species was used not only since it is considered as a good model for the study of osmoregulatory processes due to i) its availability to cope with large changes in environmental salinity, ranging from 5 to 60 ‰ [23] and ii) the current knowledge on metabolic and endocrinological aspects of osmoregulation [23–29], but also due to its importance in Spanish and European aquaculture since osmoregulatory process produces variations in the growth performance of the specimens [30]. The present work will expand the present knowledge on the tissue processes involved in fish osmoregulation, which will provide valuable information for phenotyping the adaptability of fish populations to salinity changes in productive aquaculture.

## Material and Methods

### Animals and experimental protocol

Immature males of gilthead sea bream (80–100 g body mass, n = 36) were provided by Servicios Centrales de Investigación en Cultivos Marinos (SCI-CM, CASEM, University of Cádiz, Spain; Operational Code REGA ES11028000312). Fish were transferred to the wet laboratories at the Faculty of Marine and Environmental Sciences (Puerto Real, Cádiz, Spain), where they were acclimated for 10 days to experimental control seawater (SW, 38‰ salinity, 1049 mOsm·kg^−1^ H_2_O osmolality). Fish were randomly distributed in three 400-L tanks in an open system circuit (~ 2.5 kg·m^−3^ density), under natural photoperiod (May, 2009; approximately 11 hours of light) and constant temperature (18–19°C). After the acclimation period, the animals were directly transferred, using two different tanks for each experimental condition, to the following environmental salinities: seawater (SW, 38‰ salinity, control group), low salinity water (LSW, 5‰ salinity, 139 mOsm·kg^−1^ H_2_O osmolality, hypoosmotic environment), and high salinity water (HSW, 55‰ salinity, 1439 mOsm·kg^−1^ H_2_O osmolality, hyperosmotic environment). It has been demonstrated that under these experimental conditions, gilthead sea bream specimens activate the osmoregulatory system reaching a regulatory period, where a new allostatic status is obtained after 7 days post-transfer [23, 26, 31]. The experimental salinities were achieved either by mixing SW with dechlorinated tap water for LSW, or with natural marine salt (Salina de la Tapa, El Puerto de Santa María, Cádiz, Spain) for HSW. Groups were maintained under a closed recirculation system, and at least 10% of the water volume of each tank was replaced every two days with water from a reservoir previously adjusted to the experimental salinity required. Water quality was checked twice a day to affirm their stability. Fish were fed a daily ration of 1% of their body mass with commercial pellets (Dibaq-Diproteg S.A., Segovia, Spain).

On day 7 post-transfer, 8 fish from a total of 12 specimens maintained in each experimental salinity (SW, LSW and HSW) were randomly sampled, deep-anesthetized with 2-phenoxyethanol (overdose induced with 1 mL·L^−1^ at the specific salinity water), and killed by sectioning the spinal cord. After blood extraction, hypothalamic lobules and representative biopsies of liver and gills were dissected and placed in separated eppendorf tubes, containing 600 μL of RNAlater (Life Technologies). Tubes were incubated for 24 h at 4°C and stored at −20°C afterwards.

No mortality was observed in any of the groups during experimentation. The experiment was performed according to the Guidelines of the European Union (2010/63/UE) and Spanish legislation (RD 53/2013 and law 32/2007) regarding the use of laboratory animals. The experimental procedure was authorized by the board of Experimentation on Animals of the University of Cádiz (UCA), and approved from the Ethical Committee Competent Authority (Junta de Andalucía Autonomous Government) under the reference number: 28-04-15-241.

### RNA extraction and quality analysis

Total RNA was extracted using the NucleoSpin RNA II kit (Macherey–Nagel) according to manufacturer's instructions. Tissue samples were incubated with RNase free DNase (Macherey–Nagel) during 30 min at 37°C to eliminate potential genomic DNA contamination. RNA concentrations were spectrophotometrically measured at 260 nm with the BioPhotometer Plus (Eppendorf) and RNA quality was determined using the Agilent RNA 6000 Nano Assay Kits on an Agilent 2100 Bioanalyzer (Agilent Technologies). RIN (RNA Integrity Number) values were 8–10, for almost all samples, which was indicative of clean and intact RNA to be used on microarray gene expression profiling and quantitative real-time PCR (qPCR) validation procedures. Finally, the same RNA samples from those five fish of each experimental salinity that presented the highest RIN value in the three tissues analyzed (liver, gills and hypothalamus) were individually used for both microarray hybridizations and real-time qPCR validation.

### Microarray hybridizations

The design of the oligo-microarray used in this experiment is stored in the NCBI Gene Expression Omnibus (GEO) database under accession number GPL13442. It contains 43,398 60-mer probes directed to 15,845 different transcripts, and was previously used to assess the response of cardiac and skeletal muscle tissues facing reduced nutrient availability [22]. RNA labelling, hybridizations and scanning were performed according to the manufacturer’s instructions. Briefly, total RNA (100 ng) from each individual sample was amplified and Cy3-labelled with Agilent One-Color Microarray-Based Gene Expression analysis (Low Input Quick Amp Labelling kit) along with Agilent One-color RNA SpikeIn kit. After labelling, cRNA was purified with RNeasy mini spin columns (Qiagen, Chatsworth, CA, USA) and quantity and quality was checked using a nanodrop (Thermo Scientific, Wilgminton, DE, USA) and 2100 Bioanalyzer (Agilent), respectively. Each cRNA sample (1.65 μg) was then hybridized to the gilthead sea bream array at 65°C for 17 h using the Agilent GE hybridization kit. After washing, arrays were scanned with the Agilent scanner G2505B. Spot intensities and other quality control features were extracted using the Agilent Feature Extraction software version 10.4.0.0, and were deposited in GEO under accession identifier GSE73872.

### Microarray data analysis

Microarray data were analyzed by means on the GeneSpring GX 13.0 software (Agilent). Raw data (median intensity of each spot) were extracted and corrected for background with the Agilent Feature Extraction plug-in, and then normalized using the 75^th^ percentile shift. Further statistical analysis consisted in a one-way ANOVA (corrected P value < 0.05, Tukey’s HSD post-hoc test, Benjamini-Hochberg multiple testing correction) among the nine experimental datasets (3 salinities and 3 tissues). Differentially expressed probes were then re-annotated by blast comparisons of their nucleotide sequences with those found at NCBI database and the updated version of the gilthead sea bream transcriptome nucleotide database (www.nutrigroup-iats.org/seabreamdb). Expression values of differentially expressed probes after hypoosmotic (SW→LSW) or hyperosmotic (SW→HSW) challenges that corresponded to a same annotation/gene were averaged to express the results as unigene fold-changes. Functional analysis was performed by the Ingenuity Pathway Analysis software (IPA, www.ingenuity.com), providing for each annotated sequence the Uniprot accession of the equivalent protein for model species (human, rat or mouse) as reported in [32]. Overlapping representation of canonical pathways was employed for the visualization in an integrated manner of the most responsive processes after osmotic challenges in the three analyzed tissues.

### Real-time qPCR validation

Up to 8 representative genes of significant pathways were validated by qPCR on the same individual samples used for microarray analyses (3 tissues from 5 fish maintained in 3 different experimental salinities). First, specific primer pairs of differentially expressed genes covering a wide range of variations (from ~-5.50 to ~3.50) in the microarray analysis were retrieved from [10] (SDHA and UQCRC1), [28] (ATP1A1) or designed using the software Primer3 v.0.4.0 (available in http://bioinfo.ut.ee/primer3-0.4.0/) (SULT1A1, SULT1A2, GSTA1, F11R and STMN1), and total RNA (500 ng) was reverse-transcribed in a 20 μL reaction using the qScript cDNA synthesis kit (Quanta BioSciences) as described by the supplier. To optimize the qPCR conditions, several primer concentrations (200 and 300 nM) and a temperature gradient (from 54 to 62°C) were used. In addition, different cDNA template concentrations estimated from total input of RNA were applied in triplicate (serial dilutions from 10 ng to 3.2 pg) to assess their respective assay linearity and amplification efficiencies (r^2^>0.990; efficiencies: 0.96–1.03). Finally, each reaction mixture (10 μL) contained 4 μL (10 ng) of template, 0.5 μL of each specific forward and reverse primer at their respective final concentration (Table 1), and 5 μL of PerfeCTa SYBR Green FastMix (Quanta BioSciences). Reactions were conducted in Hard-Shell PCR Plates 96-well WHT/CLR plates (Bio-Rad) covered with adhesive Microseal 'B' seal films (Bio-Rad). The thermocycling procedures were performed in a CFX Connect Real-Time system (Bio-Rad) as follows: 95°C, 10 min; [95°C, 20 s; 60°C, 30 s] x 40 cycles; melting curve [60°C to 95°C, 20 min]; 95°C, 15 s. Melting curves were used to ensure that only a single PCR product was amplified and to verify the absence of primer–dimer artefacts. Each sample was run in triplicate. The results were normalized to β-actin levels because of its abundance and Ct values consistency among tissues and treatments. Relative gene quantification was performed using the ΔΔCt method [33]. Results were expressed as mean fold-changes with respect to the control group (38‰ salinity) from each tissue and salinity, and represented in a scatter plot comparing fold-changes obtained in the microarray analysis (X-axis) against those achieved by qPCR quantification (Y-axis) in each case.

**Table 1:** Primers used for real-time qPCR validation.

Gene name	Symbol	Direction	Primer sequence (5’→3’)	Primer concentration
Succinate dehydrogenase [ubiquinone] flavoprotein subunit	SDHA	Fw	CAATCTCTGGATGAGCAGGACTGT	300 nM
		Rv	GTAGGAGCGGATGGCAGGAG	
Cytochrome b-c1 complex subunit 1, mitochondrial	UQCRC1	Fw	TGTCCTGCTGTGGTTGCTGTT	300 nM
		Rv	CGCACTCTGTTGTAGTCGGGTAG	
Cytosolic sulfotransferase 1	SULT1A1	Fw	CCACCACTCCACGACTGATTA	300 nM
		Rv	ACCATGTTGTCTTTGGCATTG	
Cytosolic sulfotransferase 2	SULT1A2	Fw	CTACCGGTCCAGTTCATACCC	300 nM
		Rv	GGCCGAAGTGGTAATAGGACA	
Glutathione S-transferase A	GSTA1	Fw	TGGAGAAGACTCCTGGTGGTT	300 nM
		Rv	GGGTAACGCTTCTCGCATAAC	
Sodium/potassium-transporting ATPase subunit alpha-1	ATP1A1	Fw	TCGTTACAGGAGTGGAAGAAGG	200 nM
		Rv	GATGTTGGCGATGATGAAGAG	
Junctional adhesion molecule A	F11R	Fw	TTGGTTCAAATGCCAGAGTTG	300 nM
		Rv	ACTCTGCCTGCATAGGGTGTT	
Stathmin	STMN1	Fw	TCCCCACTGAAGAAAAAGGAA	300 nM
		Rv	TTGTCCAGCCAAGTGTTTCAG	

## Results

### Regulation of gene expression

**Fig 1:**
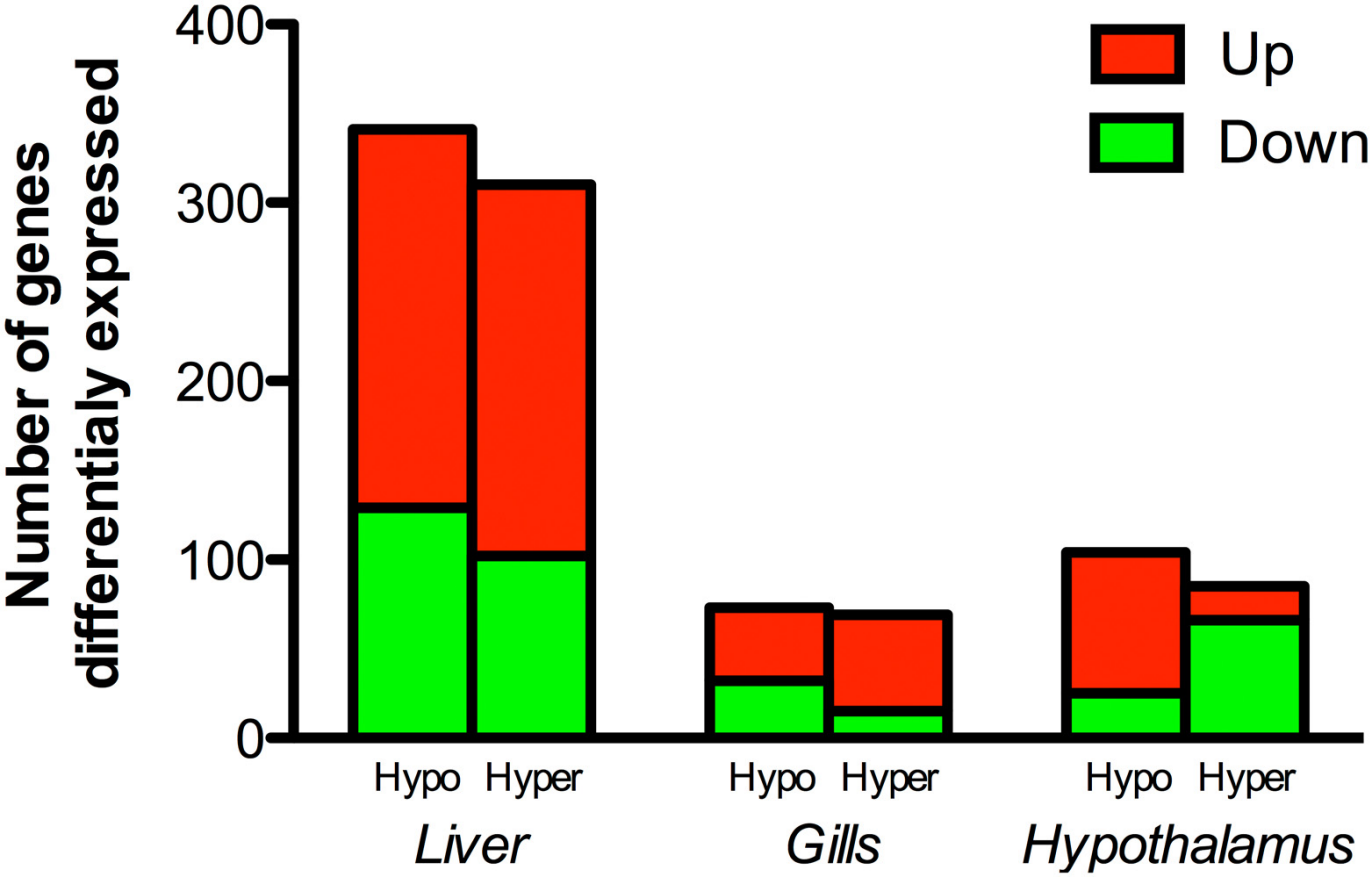
Number of up-regulated (red bars) and down-regulated genes (green bars) differentially expressed in liver, gills and hypothalamus of gilthead sea bream specimens (n = 5 fish per salinity) after 7 days of hypo- (Hypo) or hyper- (Hyper) osmotic challenge.

Number of up- (red bars) and down- (green bars) regulated unigenes that were differentially expressed in liver, gills and hypothalamus are presented in Fig 1. Liver was the most responsive tissue to salinity changes with 341 differentially expressed genes (212 up-regulated, 129 down-regulated) after hypoosmotic transfer, and 310 (208 up-regulated, 102 down-regulated) after hyperosmotic challenge. The number of differentially expressed genes in gills was reduced to 73 (41 up-regulated, 32 down-regulated) and 69 (54 up-regulated, 15 down-regulated) after hypoosmotic or hyperosmotic transfer, respectively. In the hypothalamus, the number of differentially expressed genes after hypoosmotic and hyperosmotic challenges was 104 (79 up-regulated, 25 down-regulated) and 75 (19 up-regulated, 56 down-regulated), respectively. The entire list of differentially expressed genes, including the fold-change induced by each salinity challenge (Hypo, SW → LSW; Hyper, SW → HSW) is presented for the three tissues analyzed in Table A in S1 File.

**Fig 2:**
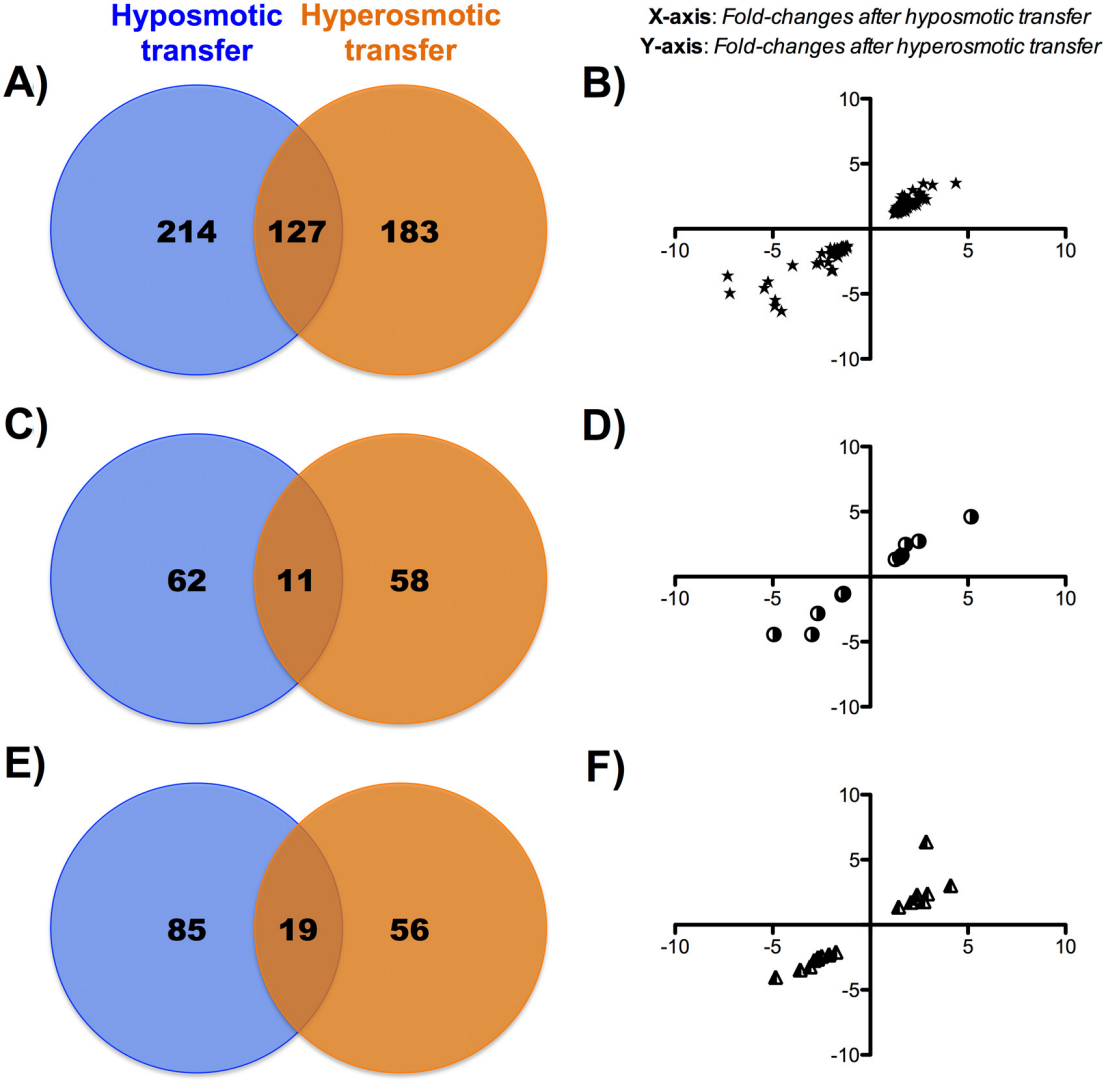
Venn diagrams of A) liver, C) gills and E) hypothalamus of gilthead sea bream specimens (n = 5 fish per salinity) showing differentially expressed transcripts that are unique to and common between hypo- or hyper-osmotic challenges 7 days post-transfer. Scatter plots in panels B), D) and F) represent fold-changes after hypo- (X-axis) and hyper- (Y-axis) osmotic transfers in common genes differentially expressed in liver, gills and hypothalamus, respectively.

When the tissue-osmoregulatory responsiveness was analyzed by Venn diagrams, the number of differentially expressed genes that were responsive either to hyper- and hypo- osmotic challenges was 127 (~ 24% of differentially expressed genes) in the liver (Fig 2A). In gills, 11 out of 131 genes (~ 8%) were differentially expressed by both hyper- and hypo- osmotic challenges (Fig 2C). Likewise, at the hypothalamic level, 19 out of 160 genes (~ 12%) were differentially expressed by hyper- and hypo- osmotic challenges (Fig 2E). Of note, in all tissues, the calculated fold-changes for these coincident genes highlights that the magnitude and direction of change (up- or down-regulation) was quite similar regardless of the hypo- or hyper- osmotic regulation needs (Fig 2B, 2D and 2F).

### Network analysis and visualization

**Fig 3:**
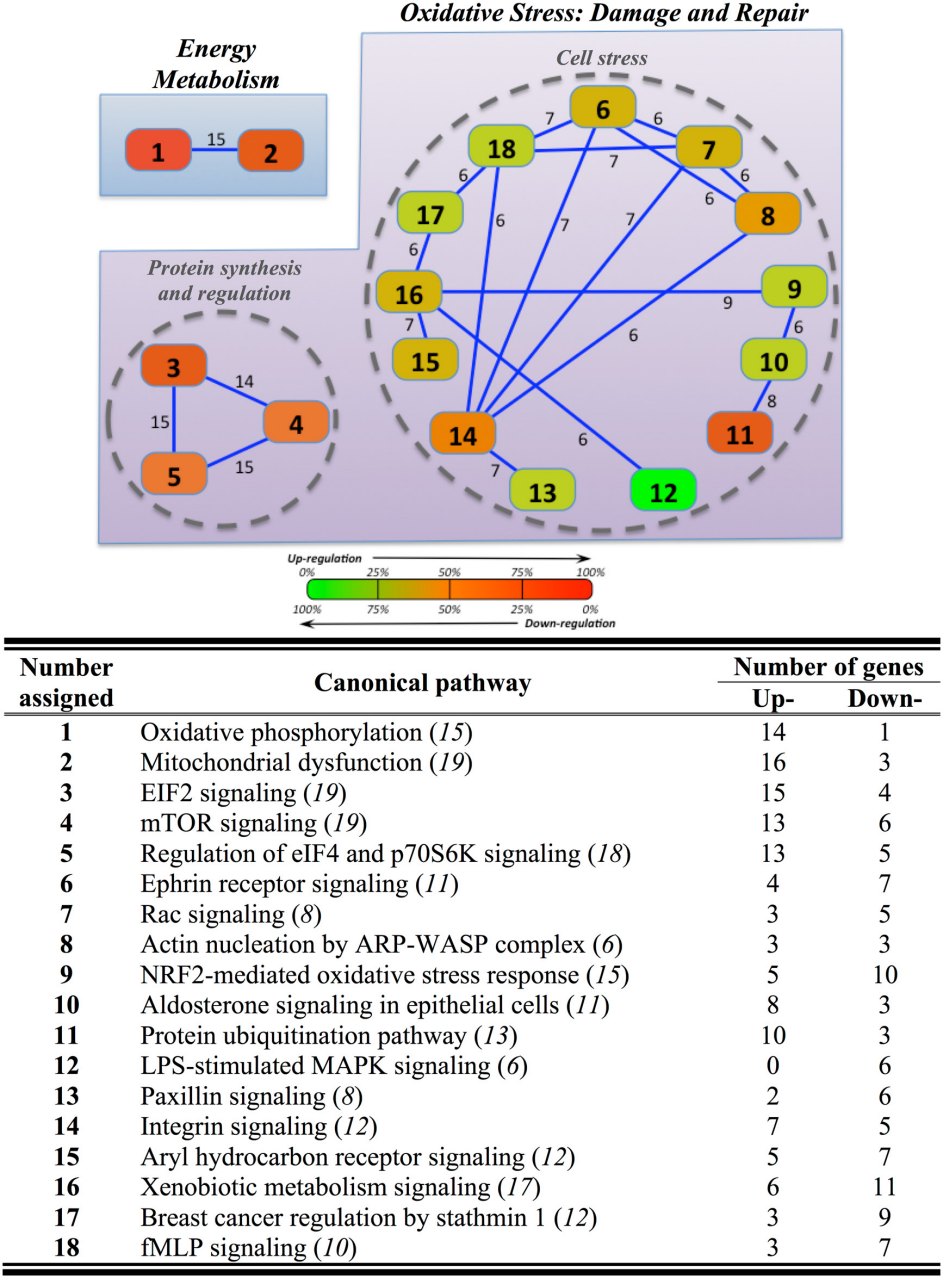
Overlapping network of hepatic response to hyper- and hypo-osmotic challenges in gilthead sea bream specimens (n = 5 fish per salinity) after 7 days post-transfer. Overlapping was generated by using Ingenuity Pathway Analysis (IPA) tools. Settings were selected to guarantee a minimum of 6 common genes between different canonical pathways. Solid lines show a direct connection between canonical pathways, indicating the number of common genes presented between them. Names and numbers assigned to each canonical pathway are represented in the table appended, indicating in brackets and italics the number of genes that compose each of them, as well as the number of up- and down-regulated genes in each canonical pathway. In each canonical pathway, a colour ranging between red (100% up-regulated) and green (100% down-regulated) was also assigned.

The functional search of osmotic-responsive genes by canonical pathway overlapping yielded a total of 91 genes in the hepatic tissue (Table B in S1 File). These genes were mapped into 18 canonical pathways, strongly interconnected by 6–15 genes each (60 overlapping genes in total) (Fig 3). In such cluster representation, two nodes named “Energy Metabolism” and “Oxidative Stress: Cell and Tissue Damage and Repair” were identified. The first node was represented by 19 mitochondria-related genes, mapped in two different pathways (oxidative phosphorylation, OXPHOS; mitochondria dysfunction) that were consistently up-regulated (16 out of 19 genes). The node of “Oxidative Stress” contained two sub-nodes with different regulatory features. The sub-node named “Protein synthesis and metabolism”, represented by mTOR signaling, EIF2 signaling-regulation of eIF4 and p70S6K signaling, was consistently up-regulated (17 out of 26). In contrast, the second sub-node so-called “Cell stress” included 13 different canonical pathways with a gradual change from up- to down-regulation. The up-regulated response was mostly related to protein ubiquination (10 out of 13 genes) pathways. The down-regulation was exemplified by the LPS-stimulated MAPK signaling (6 out of 6), whereas the response of integrin signaling (7 up-regulated and 5 down-regulated genes) and aryl hydrocarbon receptor signaling (5 up-regulated and 7 down-regulated genes) pathways was more balanced.

**Fig 4:**
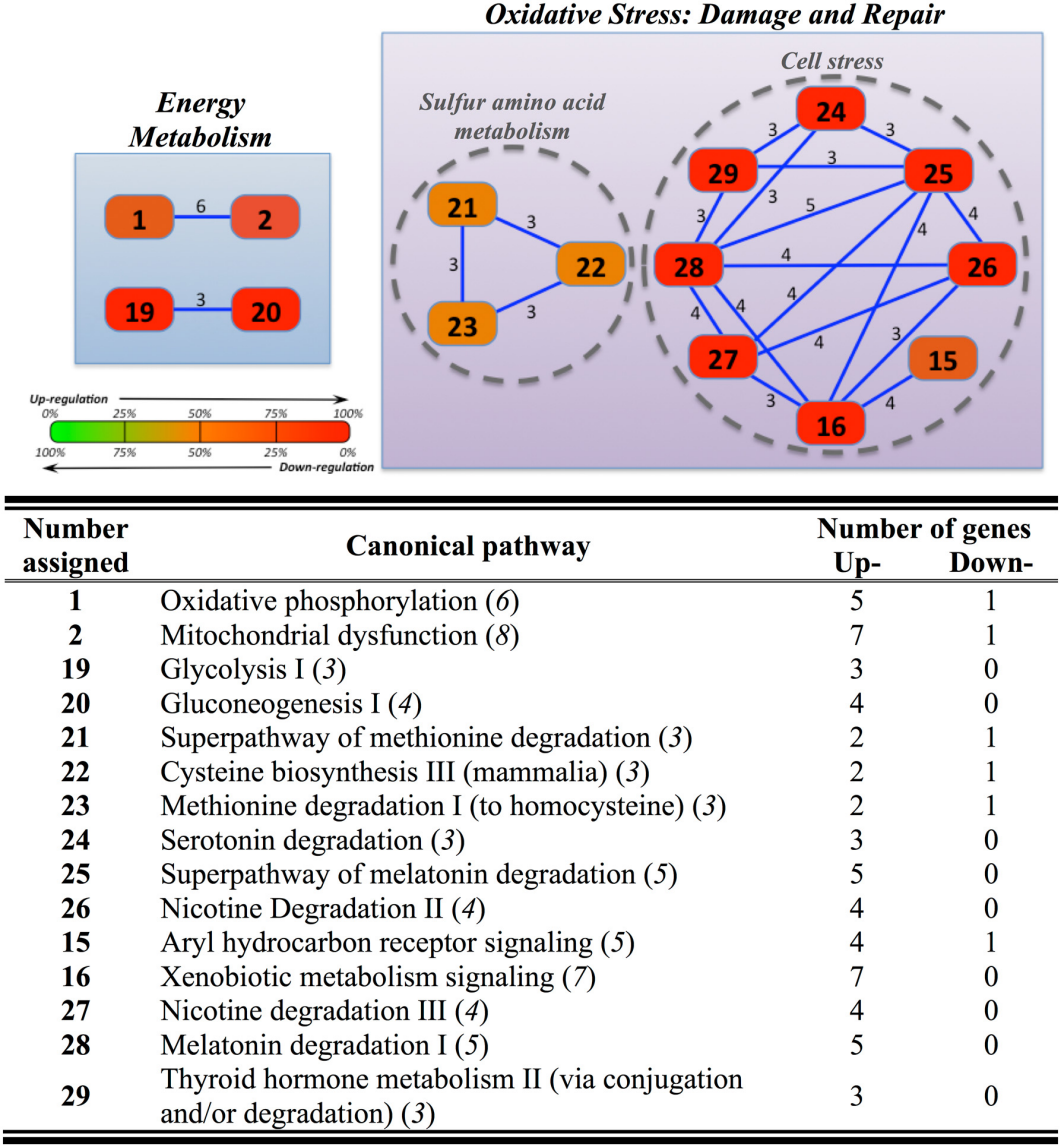
Overlapping network of branchial response to hyper- and hypo-osmotic challenges in gilthead sea bream specimens (n = 5 fish per salinity) after 7 days post-transfer. Overlapping was generated by using Ingenuity Pathway Analysis (IPA) tools. Settings were selected to guarantee a minimum of 3 common genes between different canonical pathways. For further details, see the legend of Fig 3.

The overlapping chart for gills also revealed two major nodes, equivalents to those found in the liver. This refers to a total number of 24 genes (Table C in S1 File) that were mapped into 15 canonical pathways interconnected among them by 3–6 genes (Fig 4). The node of “Energy Metabolism” included two sub-nodes related to OXPHOS and carbohydrate/anaerobic metabolism (glycolysis I, gluconeogenesis I), whereas that of “Oxidative Stress” was most represented by those canonical pathways related to i) “Sulfur amino acid metabolism” (superpathway of methionine degradation, cysteine biosynthesis III, methionine degradation I to homocysteine) and ii) “Cell stress” (serotonin degradation, melatonin degradation, nicotine degradation II, aryl hydrocarbon receptor signaling, xenobiotic metabolism signaling, nicotine degradation III, melatonin degradation I, thyroid hormone metabolism II-via conjugation and/or degradation). Regardless of gene set, the overall response was the up-regulation (21 out of 24), which was especially evident for the node “Energy Metabolism” and the sub-node “Cell stress”.

**Fig 5:**
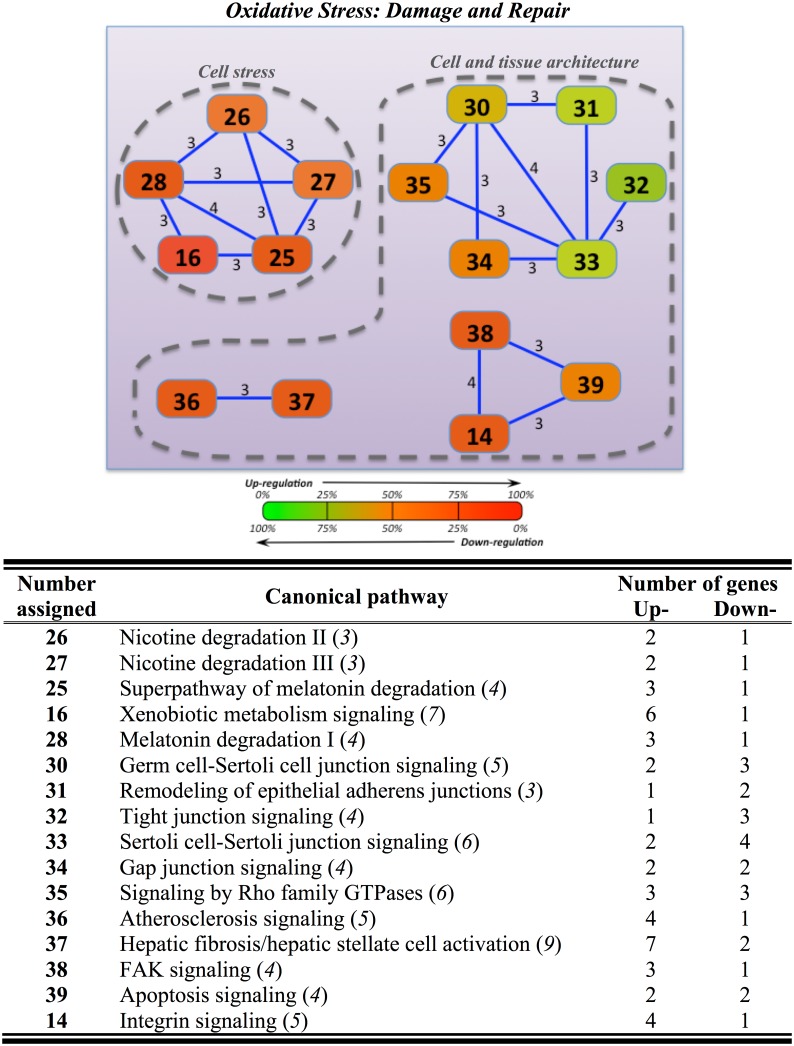
Overlapping network of hypothalamic response to hyper- and hypo-osmotic challenges in gilthead sea bream specimens (n = 5 fish per salinity) after 7 days post-transfer. Overlapping was generated by using Ingenuity Pathway Analysis (IPA) tools. Settings were selected to guarantee a minimum of 3 common genes between different canonical pathways. For further details, see the legend of Fig 3.

In the hypothalamus, up to 32 differently expressed genes were mapped into 16 overlapping canonical pathways, interconnected among them by 3–4 common genes (Table D in S1 File). This yielded two sub-nodes included in the “Oxidative Stress” node. The sub-node “Cell stress” included five different overlapping pathways (nicotine degradation II/III, superpathway of melatonin degradation, melatonin degradation I, xenobiotic metabolism signaling). The sub-node named “Cell and tissue architecture” was composed by three independent clusters: i) apoptosis-integrin signaling (apoptosis signaling, integrin signaling), ii) atherosclerosis-hepatic fibrosis (atherosclerosis signaling, hepatic fibrosis/hepatic stellate cell activation), and iii) epithelial adherent junctions and cell to cell interactions (germ cell-Sertoli cell junction signaling, remodeling of epithelial adherens junctions, tight junction signaling, Sertoli cell-Sertoli junction signaling, gap junction signaling, signaling by Rho family GTPases) (Fig 5). Almost all clusters were up-regulated, but the clusters of epithelial adherent junctions and cell to cell interactions were primarily down-regulated (8 out of 11 genes).

### Microarray validation by real-time qPCR

**Fig 6:**
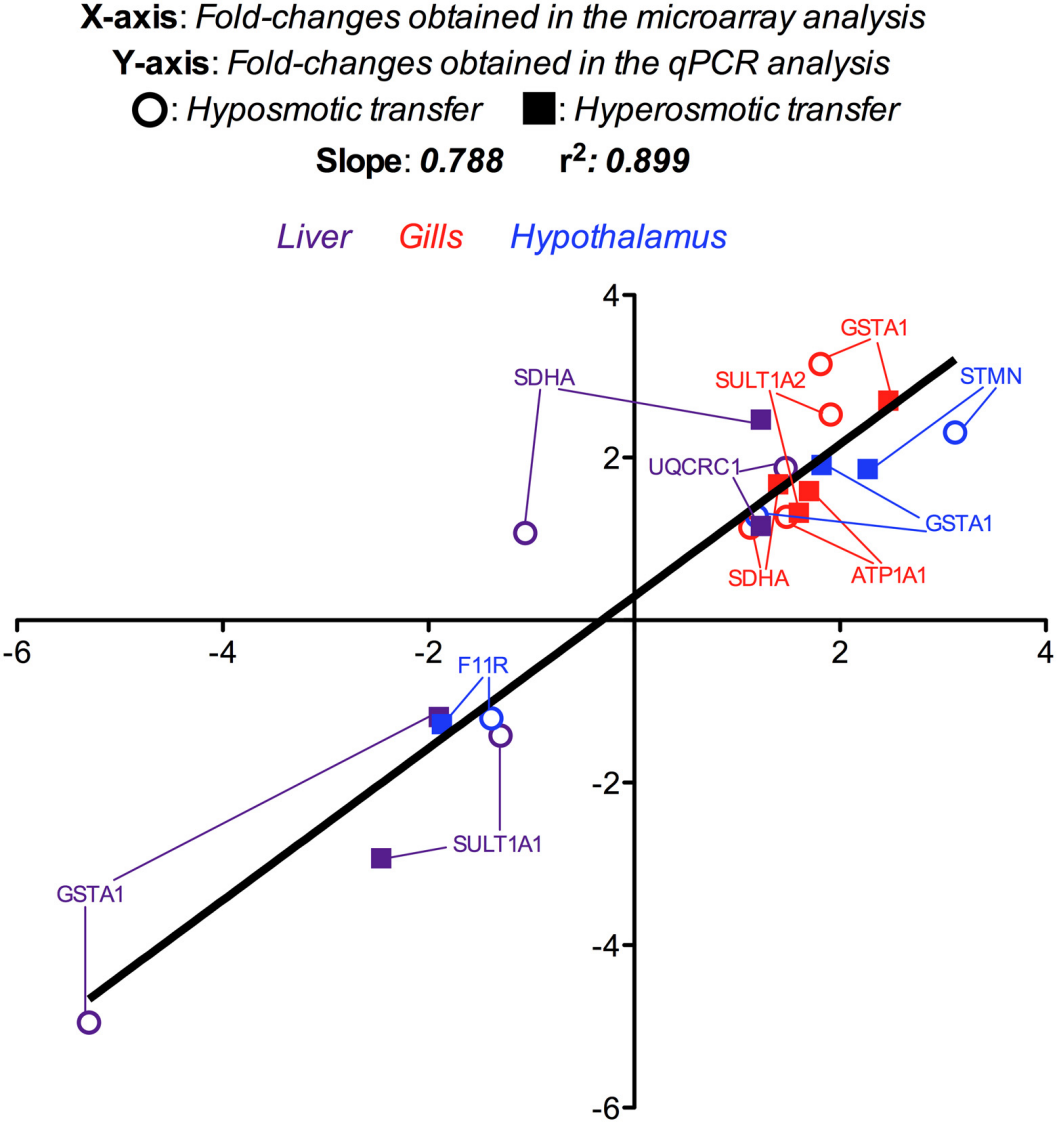
Comparison between microarray (X-axis) and qPCR (Y-axis) fold-changes for mRNA expression of differentially expressed genes used for microarray validation in liver, gills and hypothalamus of gilthead sea bream specimens transferred from seawater (SW, 38‰ salinity), to i) low salinity water (LSW, 5‰ salinity; hyposmotic challenge), and ii) high salinity water (HSW, 55‰ salinity; hyposmotic challenge), and maintained for 7 days under this osmotic conditions (n = 5 fish per salinity). SDHA: Succinate dehydrogenase [ubiquinone] flavoprotein subunit; UQCRC1: Cytochrome b-c1 complex subunit 1, mitochondrial; SULT1A1: Cytosolic sulfotransferase 1; SULT1A2: Cytosolic sulfotransferase 2; GSTA1: Glutathione S-transferase A; ATP1A1: Sodium/potassium-transporting ATPase subunit alpha-1; F11R: Junctional adhesion molecule A (KF861997); STMN: Stathmin.

Comparison of the expression results obtained for selected genes by both microarray and real-time qPCR analyses shown high levels of consistency for both up- and down-regulated genes between the two methods (r^2^ = 0.899, Pearson’s correlation analysis, Fig 6). ß–actin was not affected by salinity, presenting less than 0.10 cycles of variation between all samples in the three tissues analyzed.

## Discussion

Effects of hypo- and hyper-osmotic challenges on osmoregulatory organs, metabolism and endocrine system have been previously reported in several euryhaline teleost species [6, 7, 34–36], including gilthead sea bream [23, 24, 26, 27, 37, 38]. All of these processes have been historically studied for specific targets, from enzyme activities to histological approaches, but to our knowledge few studies have addressed in an integrated manner the transcriptional regulation of osmoregulatory and non-osmoregulatory tissues after salinity transfer [39–41]. However, it is generally accepted that the switch from hyper- to hypo-osmoregulation, or *vice versa*, is energetically expensive and it requires marked changes in the transcriptome, including those processes related to signaling pathways and remodeling of the cellular landscape and energetics [41]. In agreement with this, our data shows a total of 777 different genes that were significantly up- or down-regulated in at least one tissue and experimental condition. The up-regulation is the overall response, although different gene expression patterns (consistently validated by qPCR) were observed depending on the tissue and biological process considered. At the same time, when considering gene coincidences between hypo- and hyper- osmotic responsive genes, the magnitude and direction of changes of all of the differentially expressed genes was similar in the three studied tissues (Fig 2B, 2D and 2F; see Table A in S1 File). These findings suggest that some molecular features are part of a common response against osmotic stress regardless of the direction of change (hypo- or hyper-) in the environmental osmolality.

Network visualization of overlapping pathways also forced the identification of closely related biological processes according to the different metabolic capabilities of each tissue. Our network analysis reveals two main nodes affected by salinity challenges, represented by i) an overall up-regulation response for “Energy Metabolism” identified in both liver and gills, and ii) “Oxidative Stress: Cell and Tissue Damage and Repair” in the three analyzed tissues, but with the most evident response to its up-regulation in gills and hypothalamus. Even so, they present different sub-nodes, which are characterized by several pathways and differentially expressed genes responding in a singular way depending on the main cellular process needed. At the same time, the depletion of some physiological processes can be viewed as an adaptive response to prime the expression of genes that really play a feedback role after salinity transfer.

The energy cost of osmoregulation is greatly variable, but it is now accepted that around 10 to 50% of the total fish energy budget is dedicated to osmoregulation [42]. Indeed, the gills present an important energy expenditure [43, 44]. The liver cannot be considered a really osmoregulatory tissue, although it is contemplated as one of the most important energy suppliers to fish osmoregulatory tissues [7, 45], due to its critical significance in the metabolism of carbohydrates and lipids [46]. Accordingly, it is important to remark the consistent activation of energy-generation processes in both hepatic and branchial tissues. In liver, OXPHOS and mitochondrial dysfunction canonical pathways denote this metabolic feature. These pathways included genes related with ATP and NADH production (ATP synthases, 2-oxoglutarate dehydrogenase) or respiratory electron transport chain (cytochrome b-c1 complex and cytochrome c oxidase subunits, succinate dehydrogenase [ubiquinone] subunits), which mRNA expression might became enhanced by osmotic transfers to cope with increased cell and tissue energy needs. Similarly, all the genes involved in branchial energy production, encompassed within four different canonical pathways (OXPHOS, mitochondrial dysfunction, glycolysis I, gluconeogenesis I), were up-regulated in both hypo- and hyper- osmotic transfers, excluding the ATP synthase-coupling factor 6 (mitochondrial) (ATP5J), which was down-regulated after hypoosmotic challenge. Coupling factor 6 is an essential component of the energy-transducing stalk of mitochondrial ATP synthase [47], but also acts in vascular endothelial cells inducing vasoconstriction [48]. According with this hint, the down-regulation of this gene in fish acclimated to hypoosmotic environment suggests that ATP5J decreases the vasoconstriction function in gills where lower rates of ion loss are required. Certainly, higher rates in active ion exchange and water movement produced by key transporters and channels in mitochondrion-rich cells (also called chloride cells) of the branchial tissue are required in response to osmotic stress (see [49] for review). In the present study, this notion was supported by the clear activation of two important ion transporters. First, Na^+^,K^+^-ATPase (ATP1A1) significantly increased after hyperosmotic transfer showing the typical “U-shape” relationship previously observed in gilthead sea bream after osmoregulatory responses both at transcriptional (Fig 6; Table A in S1 File; [28]) and enzymatic activity [23] levels. Secondly, branchial carbonic anhydrase (CA) clearly increased after hypoosmotic challenge (Table A in S1 File), further corroborating that this enzyme is involved not only in CO_2_ excretion but also in osmoregulatory process [50, 51] in response to low-salinity exposure, operating at either the transcriptional or translational level [52]. Even so, energy metabolism did not appear to be enhanced on the hypothalamus tissue by osmotic stress. Probably, this finding reflected a high metabolic resilience of the central nervous system, which is at the same time indicative of its high robustness to preserve a given metabolic and phenotype condition when facing changes in environmental and nutritional cues [53, 54].

Markers of “Oxidative Stress” included in the sub-node “Cell stress” were over-represented within the full list of differentially expressed genes regardless of tissue and type of osmotic stress. Hence, a common feature of each tissue flowchart of overlapping canonical pathways of differentially expressed genes was xenobiotic metabolism. This finding discloses that biochemical modifications carried out through specific enzymatic systems are needed to produce readily excreted products under adverse metabolic situations with an increased risk of oxidative stress [55]. Herein, this important function is highlighted by the up-regulation of a number of cytochromes (i.e. P450 and several complex subunits) in all the analyzed tissues after both salinity transfers, indicating that important metabolic modifications occur to cope with the fish osmolite misbalances. However, other closely-related processes (LPS-stimulated MAPK signaling, NRF2-mediated oxidative stress response) were at the same time markedly down-regulated in the liver tissue. Importantly, LPS (lipopolysaccharide) is a component of the cell wall of Gram-negative bacteria. These molecules regulate the expression of pro-inflammatory cytokines and adhesion molecules that contribute to the pathogenesis of septic shock [56]. To our knowledge, few studies have addressed the regulation of the immune response after salinity transfer in fish [57], but the depletion of LPS response pathway in our experimental model might be indicative of a cellular response of immuno-competence due to the osmoregulatory disorder. This fact may facilitate at short-term the sparing of metabolic fuels for osmoregulation to canalize this energy budget to other important processes required.

Functional analysis of differentially expressed genes also highlighted that xenobiotic metabolism was co-regulated with melatonin-related genes. Melatonin hormone is secreted by the pineal gland and it is an important player in several functions related to osmoregulation [58–60], but also to interact with other well-known osmoregulatory fish hormones produced in the hypothalamus, like arginine vasotocin or isotocin [61, 62]. In addition, salinity transfer promoted thyroid hormone metabolism in peripheral tissues (e.g. gills) of teleosts, including the gilthead sea bream, being both sulfation (catalyzed by sulfotransferases) and glucuronidation (catalyzed by UDP-glucuronosyltransferases) processes, which are clearly activated in our experimental approach, important players in its regulation [63–65]. These insights confirm the great importance of this peripheral metabolism after osmotic challenges also in gills, demonstrating a huge significance of the endocrine cascade in the regulation of the osmoregulatory functions.

Within the liver, even with the dualism for up- and down-regulated overlapping canonical pathways (sub-node “Cell stress”), protein ubiquitination and integrin signaling pathways mostly exhibited an up-regulated response. Ubiquitination regulates degradation of cellular proteins, controlling a protein half-life and expression levels. This pathway was represented, among other genes, by different heat shock proteins (HSP). The HSPs (10 kDa HSP, 60 kDa HSP, 70 kDa HSP, 90 kDa HSP) were up-regulated by hypoosmotic challenges in liver and also in gills. The same pattern has been recently reported in the blue-green damselfish (*Chromis viridis*) in response to reduced salinity [66], illustrating that HSP system and ubiquitination are important pathways to prevent osmotic stress damage for maintaining cellular protein quality. In turn, integrins are transmembrane molecules responsible for cell-cell and cell-extracellular matrix interactions [67]. Our results showed that different genes involved in this canonical pathway (e.g. different actin-related proteins, calpain-1 catalytic subunit, Ras-related protein Rap-1, or vinculin) were triggered in liver by the osmotic challenge, resulting in an up-regulation of the cell cycle and the signal transduction [68]. In fact, calpain-1 was found to increase its mRNA expression after starving conditions to improve protein mobilization as a source of energy in rainbow trout (*Oncorhynchus mykiss*) fingerlings [69], whereas Ras-related protein Rap-1 has been also suggested to be regulated by osmotic stress [70]. In this regard, a preferential up-regulation in the sub-node involved in “Protein synthesis and metabolism” is also observed. Moreover, “Sulfur amino acid metabolism” sub-node identified in gills showed the same up-regulatory pattern after both salinity transfers, confirming the nitrogenous turnover, where two essential amino acid pathways (methionine and cysteine) were affected at transcriptional level, also in this important osmoregulatory tissue after osmotic stress.

In branchial and hypothalamic tissues, a clear up-regulated interconnection in the pathways composing the sub-node “Cell stress” was evidenced (Figs 4 and 5). Indeed, in gills, only the apoptotic BAX gene was down-regulated by hypoosmotic challenge. This gene accelerates programmed cell death causing translocation to the mitochondrion membrane, and leads to the release of cytochrome c that then triggers apoptosis [71]. Recent studies confirm that mRNA expression of branchial BAX gene is up-regulated after hyperosmotic challenges in the climbing perch (*Anabas testudineus*). These environments are more likely to produce apoptosis involving the removal of certain types of branchial cells [72], which may be as a consequence of a higher chloride cells need to deal with the osmoregulatory load (see above; [23]). In the same way, all genes from canonical pathways related to this sub-node in hypothalamus were up-regulated, except the UDP-glucuronosyltransferase 1–9 (UDPGT1-9). The down-regulation observed in this gene could be due to its specific function in phenols and chemical compound elimination [73, 74] and the presumably absence of them as pollutants in our facilities.

Functional analysis of differentially expressed genes also highlighted an exclusive hypothalamic sub-node named “Cell and tissue architecture”, which was mostly down-regulated (72% of the genes) after both salinity transfers (Fig 5). This down-regulation suggests that hypothalamic cells tend to reduce tight junctions, giving that large protein molecules, as well as solutes and water, move freely across the tissue [75], facilitating their arrival to the hypothalamic nuclei where they are required [76]. Changes in plasma tonicity are clinically used to increase blood-brain barrier permeability [77] in order to deliver different substances to the central nervous system [78]. Interestingly, other two genes involved in cell junction signaling, i.e. actin and dual specificity mitogen-activated protein kinase kinase 2 (MAP2K2), were up-regulated after hypoosmotic acclimation. This co-regulated response of genes encoding actin and collagen chains could be needed to increase cell volume in response to the decrease in plasma osmolality after hypoosmotic transfer, which has been described in several teleost species including the gilthead sea bream [23, 79–81]. Taking together with the effect observed in tight cell junctions, it is strongly suggested that large responses for the low solute and water permeability of cerebral capillaries were needed [75]. Moreover, increased MAPK gene expression indicates that a higher regulation of common cellular functions, including metabolic, osmoregulatory and endocrine pathways [82–84], as well as in the regulatory volume increase process [85], is controlled by this mechanism and required to overcome these osmotic challenges.

## Concluding Remarks

**Fig 7:**
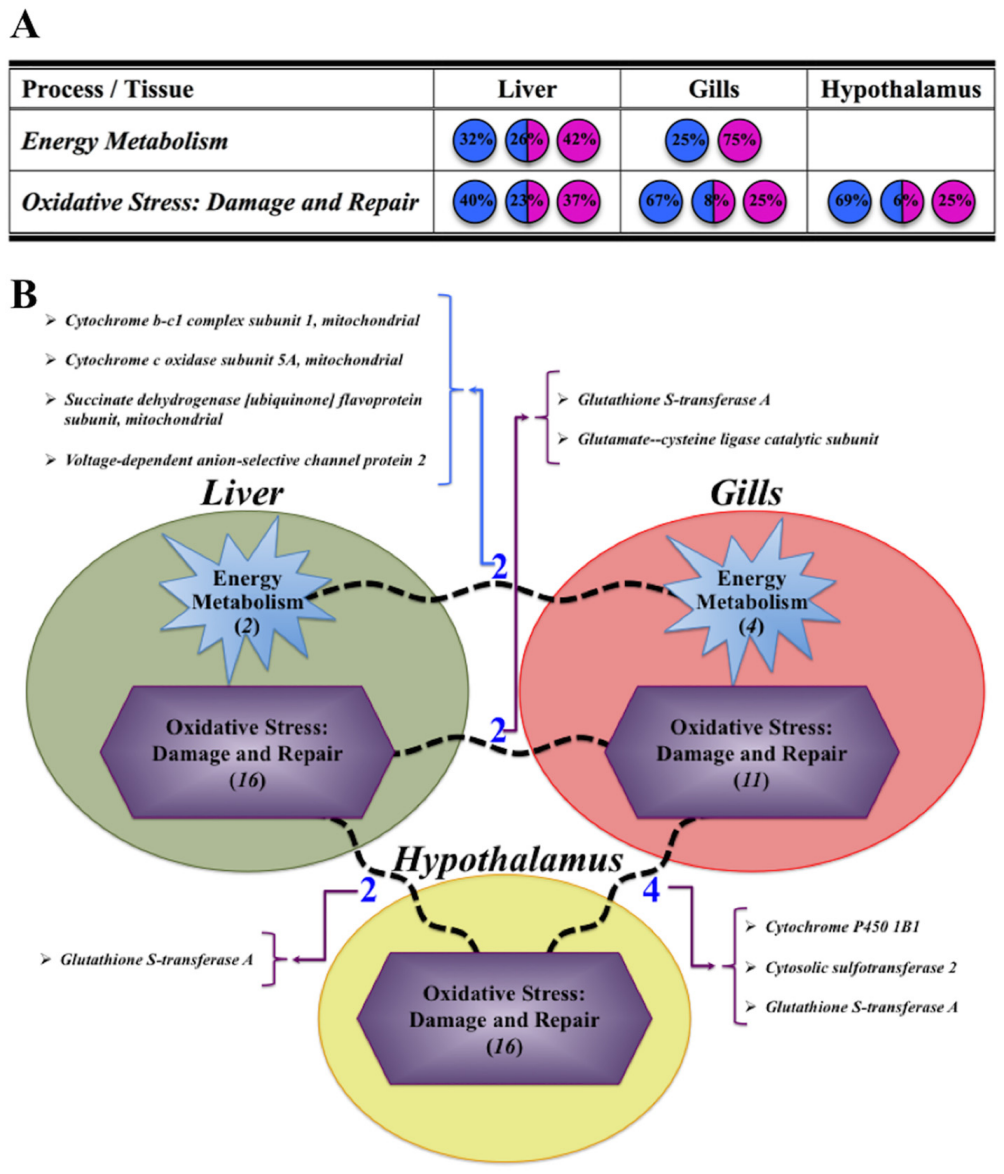
(A) Evaluation of main processes detected in liver, gills and hypothalamus, with indication of the percentage (%) of genes differentially expressed and regulated by each salinity transfer (Hypo: blue, Hyper: pink or both: blue/pink). (B) Schematic representation of those clusters identified in each studied tissue, indicating and naming the number of common pathways (broken lines) and genes shared by them. Number of total pathways present in each cluster is displayed in brackets and italics.

All together, the results presented herein indicate that the hepatic, branchial and hypothalamic transcriptomes of gilthead sea bream are highly regulated after 7 days of transfer to different environmental salinities. Importantly, our findings clearly indicate that a well-defined duality between physiological processes and their responses in liver and gills (Energy Metabolism and nitrogenous turnover) or in gills and hypothalamus (cell stress) can be specifically considered after hypo- or hyper-osmotic conditions. At the same time, hypothalamus seems to be a particular tissue, and the osmotic adaptive response was mostly mediated by cell and tissue architecture genes in order to preserve its specific allostatic condition. All these findings with specific indication of common genes among shared pathways are summarized in Fig 7 for a comprehensive overview of adaptive stress osmoregulation at the transcriptional level. These findings would be taken into account in future studies in order to phenotype fish strains with greater potential of adaptation to higher or lower salinity environments.

## Supporting Information

S1 File Table A in S1 File. List of differentially expressed genes in the three analysed tissues. Table B in S1 File. Differentially expressed genes mapped in overlapping pathway charts of liver. Table C in S1 File. Differentially expressed genes mapped in overlapping pathway charts of gills. Table D in S1 File. Differentially expressed genes mapped in overlapping pathway charts of hypothalamus.
(DOCX)
