# Supplementary material for: Unraveling the Tissue-Specific Gene Signatures of Gilthead Sea Bream (Sparus aurata L.) after Hyper- and Hypo-Osmotic Challenges
Source: PLoS One. 2016 Feb 1;11(2):e0148113. doi: 10.1371/journal.pone.0148113 (PMC4734831; doi:10.1371/journal.pone.0148113)
Supplement: S1 File — Table A in S1 File. List of differentially expressed genes in the three analysed tissues. Table B in S1 File. Differentially expressed genes mapped in overlapping pathway charts of liver. Table C in S1 File. Differentially expressed genes mapped in overlapping pathway charts of gills. Table D in S1 File. Differentially expressed genes mapped in overlapping pathway charts of hypothalamus. (DOCX) [file pone.0148113.s001.docx]

**S1 File Table A. List of differentially expressed genes (P<0.05, one-way ANOVA with corrected P<value, Tukey´s HSD post hoc test, Benjamini-Hochberg multiple testing correction) with fold-change values after hypo- (Hypo) and hyper- (Hyper) transfer referred to seawater acclimated fish (control, 38 ppt).**

| **Gene Name** | **Fold-Change** | |
| --- | --- | --- |
|  | **Hypo** | **Hyper** |
| ***Liver*** |  |  |
| [Pyruvate dehydrogenase [lipoamide]] kinase isozyme 3, mitochondrial | 1.48 |  |
| 1,2-dihydroxy-3-keto-5-methylthiopentene dioxygenase | 1.26 |  |
| 10 kDa heat shock protein, mitochondrial (JX975224) | 2.72 | 3.48 |
| 116 kDa U5 small nuclear ribonucleoprotein component | 1.71 |  |
| 14-3-3 protein epsilon |  | 1.42 |
| 14-3-3 protein zeta |  | 1.33 |
| 2-hydroxyacyl-CoA lyase 1 | -1.59 |  |
| 2-oxoglutarate dehydrogenase, mitochondrial | 1.43 |  |
| 28S ribosomal protein S15, mitochondrial | 1.44 | 1.36 |
| 28S ribosomal protein S17, mitochondrial | 1.46 | 1.51 |
| 28S ribosomal protein S30, mitochondrial | 1.51 | 1.70 |
| 3-hydroxyisobutyrate dehydrogenase, mitochondrial | 1.30 |  |
| 39S ribosomal protein L16, mitochondrial | 1.49 | 1.43 |
| 39S ribosomal protein L30, mitochondrial | 1.35 | 1.50 |
| 39S ribosomal protein L33, mitochondrial |  | 1.39 |
| 39S ribosomal protein L51, mitochondrial | 1.45 |  |
| 4-aminobutyrate aminotransferase, mitochondrial | -1.85 |  |
| 40S ribosomal protein S11 | 1.46 | 1.68 |
| 40S ribosomal protein S12 | 1.49 |  |
| 40S ribosomal protein S16 | 1.56 |  |
| 40S ribosomal protein S2 | 1.56 |  |
| 40S ribosomal protein S24 | 1.49 |  |
| 5'-AMP-activated protein kinase subunit beta-1 |  | -1.94 |
| 5'-nucleotidase domain-containing protein 2 | -2.55 | -2.57 |
| 55 kDa erythrocyte membrane protein | -1.63 |  |
| 6-phosphofructo-2-kinase/fructose-2,6-biphosphatase 4 | 1.73 | 2.46 |
| 60 kDa heat shock protein, mitochondrial |  | 2.80 |
| 60S ribosomal protein L10a | 1.45 |  |
| 60S ribosomal protein L13a (Fragment) | -1.92 | -3.18 |
| 60S ribosomal protein L23a | 1.76 |  |
| 60S ribosomal protein L29 | 1.97 |  |
| 60S ribosomal protein L32 | 1.49 |  |
| 60S ribosomal protein L7a | 1.41 |  |
| 60S ribosomal protein L9 | 2.18 | 1.92 |
| 90kDa heat shock protein beta (KM522803) | 1.55 |  |
| Absent in melanoma 1 protein | 1.53 |  |
| Actin-related protein 2/3 complex subunit 4 |  | 1.19 |
| Actin-related protein 2/3 complex subunit 5 | 1.37 | 1.47 |
| Actin-related protein 3 |  | 1.35 |
| Acyl-CoA desaturase (SCD1a) (JQ277703) | -2.26 |  |
| Acyl-CoA desaturase (SCD1b) | -7.30 | -3.61 |
| **Gene Name** | **Fold-Change** | |
|  | **Hypo** | **Hyper** |
| ***Liver*** |  |  |
| Acylamino-acid-releasing enzyme |  | 2.07 |
| Adiponectin receptor protein 1 |  | -1.33 |
| ADP-ribosylation factor 1 |  | 1.33 |
| Adrenodoxin, mitochondrial | -1.72 |  |
| AFG3-like protein 2 |  | 1.35 |
| Alanyl-tRNA editing protein Aarsd1 | 1.94 | 2.23 |
| Alanyl-tRNA synthetase, cytoplasmic | 1.62 |  |
| Aldehyde dehydrogenase family 3 member B1 | -2.15 | -2.59 |
| Alkylated DNA repair protein alkB homolog 1 | 1.49 | 1.55 |
| Alpha-aminoadipic semialdehyde synthase, mitochondrial | -1.50 |  |
| Alpha-enolase | -5.11 |  |
| Amyloid beta A4 precursor protein-binding family A member 1 | -1.49 | -1.57 |
| Annexin A11 | -1.48 |  |
| Apolipoprotein L3 |  | -2.16 |
| Apoptosis-associated speck-like protein containing a CARD | 2.16 |  |
| Apoptosis-inducing factor 3 | 2.35 |  |
| Arachidonate 5-lipoxygenase-activating protein | -2.24 |  |
| Aryl hydrocarbon receptor nuclear translocator-like protein 1 | -1.46 |  |
| Aspartyl-tRNA synthetase, cytoplasmic | 1.41 | 1.26 |
| Ataxin-7-like protein 3 | 2.17 | 2.97 |
| ATP synthase mitochondrial F1 complex assembly factor 2 | 1.31 | 1.38 |
| ATP synthase subunit d, mitochondrial | 1.90 |  |
| ATP-dependent DNA helicase Q4 |  | 2.70 |
| Aurora kinase A-interacting protein | 1.78 |  |
| B-cell receptor-associated protein 29 | 1.25 |  |
| Beta-1,3-galactosyl-O-glycosyl-glycoprotein beta-1,6-N-acetylglucosaminyl-transferase 7 | 2.73 |  |
| Beta-galactosidase | 1.47 |  |
| BRCA1-A complex subunit BRE | -1.36 |  |
| Bromodomain adjacent to zinc finger domain protein 2B | -1.36 |  |
| BSD domain-containing protein 1 | -1.51 |  |
| BTB/POZ domain-containing protein kctd15 |  | 1.75 |
| Bystin |  | 1.63 |
| C-X-C chemokine receptor type 7 |  | -1.82 |
| C2 domain-containing protein 3 |  | 1.59 |
| CAAX prenyl protease 1 homolog |  | 1.48 |
| Calcium uniporter regulatory subunit MCUb, mitochondrial |  | -3.30 |
| Calcium uptake protein 3, mitochondrial | -3.25 |  |
| Calcium-binding mitochondrial carrier protein Aralar1 |  | 4.27 |
| Calpain-1 catalytic subunit | 1.81 |  |
| Calpastatin | -1.50 |  |
| Calumenin | -4.87 | -5.47 |
| Carbonyl reductase [NADPH] 1 | -1.80 |  |
| Carboxypeptidase E | -3.11 |  |
| Carnitine O-palmitoyltransferase 2, mitochondrial |  | 1.46 |
| Casein kinase II subunit beta |  | 1.29 |
| Catalase (CPT1A) (JQ308823) | -1.69 | -1.66 |
| **Gene Name** | **Fold-Change** | |
|  | **Hypo** | **Hyper** |
| ***Liver*** |  |  |
| Cathepsin D | -2.69 |  |
| CDGSH iron-sulfur domain-containing protein 2A |  | 2.15 |
| Cell cycle checkpoint control protein RAD9A | 1.93 |  |
| Cell division cycle and apoptosis regulator protein 1 |  | -1.86 |
| Centrosomal protein of 290 kDa (Fragment) |  | 1.39 |
| Charged multivesicular body protein 1a | -1.35 |  |
| Choline kinase alpha | -2.05 | -1.48 |
| Choline transporter-like protein 1 |  | -1.71 |
| Cholinesterase |  | 1.88 |
| Cleavage and polyadenylation specificity factor subunit 5 |  | 1.53 |
| CLIP-associating protein 1 | -1.40 |  |
| CMP-N-acetylneuraminate-beta-galactosamide-alpha-2, 3-sialyltransferase 4 |  | -1.94 |
| Coagulation factor XI |  | -2.10 |
| Cohesin subunit SA-2 |  | -1.63 |
| Coiled-coil domain-containing protein 124 |  | 1.94 |
| Coiled-coil domain-containing protein 51 | 1.85 |  |
| Coiled-coil-helix-coiled-coil-helix domain-containing protein 7 | 1.36 | 1.31 |
| Cold-inducible RNA-binding protein A |  | -1.40 |
| Complement factor I |  | -2.57 |
| Consortin |  | -3.12 |
| Coxsackievirus and adenovirus receptor homolog |  | -2.08 |
| Cryptochrome-1 | 1.87 | 2.04 |
| CST complex subunit TEN1 | 2.38 | 2.15 |
| Cullin-1 |  | -1.37 |
| Cullin-4A |  | -1.43 |
| Cyclin-dependent kinase 2 |  | 2.21 |
| Cyclin-dependent kinases regulatory subunit 1 | -3.15 |  |
| Cyclin-Y-like protein 1 | 2.90 |  |
| Cytochrome b-c1 complex subunit 1, mitochondrial | 1.47 |  |
| Cytochrome b-c1 complex subunit 10 | 1.59 |  |
| Cytochrome b-c1 complex subunit 2, mitochondrial |  | 1.33 |
| Cytochrome b-c1 complex subunit 6, mitochondrial | 1.70 | 1.77 |
| Cytochrome b-c1 complex subunit Rieske, mitochondrial |  | 1.50 |
| Cytochrome c | 2.86 | 2.26 |
| Cytochrome c oxidase assembly protein COX11, mitochondrial |  | 1.43 |
| Cytochrome c oxidase subunit 5A, mitochondrial | 2.47 | 2.71 |
| Cytochrome c oxidase subunit 8A, mitochondrial | 1.53 | 1.45 |
| Cytohesin-4 | 2.53 |  |
| Cytosol aminopeptidase |  | 1.35 |
| Cytosolic sulfotransferase 1 |  | -2.46 |
| Delta-1-pyrroline-5-carboxylate dehydrogenase, mitochondrial |  | 1.34 |
| Delta-like protein 4 | 1.53 |  |
| DENN domain-containing protein 4C | -1.52 | -1.48 |
| Diamine acetyltransferase 2 | 3.59 |  |
| Dihydrolipoyllysine-residue succinyltransferase component of 2-oxoglutarate dehydrogenase complex, mitochondrial | 1.77 | 1.51 |
| Disks large-associated protein 4 | -1.86 |  |
| **Gene Name** | **Fold-Change** | |
|  | **Hypo** | **Hyper** |
| ***Liver*** |  |  |
| Disrupted in renal carcinoma protein 2 | -1.87 | -1.91 |
| DNA damage-inducible transcript 4 protein |  | 2.35 |
| DNA mismatch repair protein Msh2 |  | 1.71 |
| DNA replication licensing factor mcm2 |  | 2.93 |
| DNA-binding protein RFXANK | 2.02 | 2.21 |
| DNA-directed RNA polymerase I subunit RPA12 | 1.38 |  |
| DNA-directed RNA polymerases I and III subunit RPAC2 | 2.01 | 2.15 |
| DNA-directed RNA polymerases I, II, and III subunit RPABC2 |  | 1.44 |
| DnaJ homolog subfamily C member 11 | 1.55 | 2.33 |
| DnaJ homolog subfamily C member 17 | 1.40 | 1.43 |
| DnaJ homolog subfamily C member 30 | 1.33 | 1.31 |
| DnaJ homolog subfamily C member 8 |  | 1.34 |
| Dolichol phosphate-mannose biosynthesis regulatory protein |  | 1.74 |
| Dynein heavy chain 9, axonemal | -2.50 |  |
| E3 ubiquitin-protein ligase BRE1B | 1.49 | 1.71 |
| E3 ubiquitin-protein ligase listerin |  | 1.31 |
| E3 ubiquitin-protein ligase NEDD4-like |  | -1.96 |
| E3 ubiquitin-protein ligase RING2 |  | -1.57 |
| E3 ubiquitin-protein ligase TTC3 | -1.66 | -2.13 |
| ELAV-like protein 1 |  | 1.52 |
| Electron transfer flavoprotein-ubiquinone oxidoreductase, mitochondrial | -1.59 |  |
| Elongation factor 1-alpha, somatic form |  | 5.91 |
| Elongation factor 1-beta | 1.64 |  |
| Elongation factor G, mitochondrial |  | 1.74 |
| Elongation of very long chain fatty acids protein 5 | -3.92 |  |
| Endonuclease 8-like 1 |  | 2.46 |
| Endophilin-A1 | 2.76 |  |
| Endothelin-converting enzyme 2 |  | 3.16 |
| Ephrin-B1 |  | -2.38 |
| Epsilon-sarcoglycan | -1.84 | -1.49 |
| ERO1-like protein alpha | 2.47 | 2.47 |
| Estrogen receptor |  | -2.48 |
| Eukaryotic initiation factor 4A-I | 1.43 |  |
| Eukaryotic translation initiation factor 2 subunit 2 |  | 1.52 |
| Eukaryotic translation initiation factor 3 subunit A | 1.58 |  |
| Eukaryotic translation initiation factor 3 subunit F | 1.64 |  |
| Eukaryotic translation initiation factor 3 subunit K | 1.72 |  |
| Eukaryotic translation initiation factor 3 subunit L | 1.59 |  |
| Eukaryotic translation initiation factor 5A-2 | 1.52 |  |
| Exosome complex component CSL4 | 1.76 |  |
| Exosome complex component RRP46 |  | 1.64 |
| F-box only protein 8 |  | -1.96 |
| F-box/LRR-repeat protein 14 | 1.42 |  |
| Fatty acid-binding protein, heart | -1.79 |  |
| FCH domain only protein 2 | -1.29 |  |
| G protein pathway suppressor 2 | -1.62 |  |
| G/T mismatch-specific thymine DNA glycosylase | 2.04 |  |
| **Gene Name** | **Fold-Change** | |
|  | **Hypo** | **Hyper** |
| ***Liver*** |  |  |
| Gamma-glutamyl hydrolase |  | -3.45 |
| Gamma-glutamyltransferase 7 | 4.22 |  |
| GATA zinc finger domain-containing protein 1 | 1.26 | 1.28 |
| GDP-Man:Man(3)GlcNAc(2)-PP-Dol alpha-1,2-mannosyltransferase | -1.38 |  |
| Glutamate--cysteine ligase catalytic subunit |  | 1.70 |
| Glutaminyl-peptide cyclotransferase | 1.67 |  |
| Glutaryl-CoA dehydrogenase, mitochondrial |  | -1.67 |
| Glutathione S-transferase A | -5.30 |  |
| Glutathione S-transferase Mu 3 | -1.49 | -1.66 |
| Glycine dehydrogenase [decarboxylating], mitochondrial | -2.00 | -3.19 |
| Glycylpeptide N-tetradecanoyltransferase 1 |  | 1.63 |
| Group XV phospholipase A2 (JX975706) |  | 2.14 |
| Group XVI phospholipase A2 | -2.00 |  |
| Growth inhibition and differentiation-related protein 88 homolog | -1.22 |  |
| GTP-binding protein Rheb | -2.05 |  |
| GTPase IMAP family member 4 | -2.78 |  |
| GTPase KRas | -1.54 | -1.56 |
| Guanine nucleotide-binding protein G(i) subunit alpha-2 | -1.20 | -1.45 |
| Guanine nucleotide-binding protein subunit beta-4 | -1.25 | -1.30 |
| H/ACA ribonucleoprotein complex subunit 2-like protein | 1.97 | 2.03 |
| HD domain-containing protein 2 |  | 2.99 |
| Heat shock 70 kDa protein 14 | 1.53 |  |
| Heat shock cognate 70 kDa protein | 1.87 | 1.72 |
| Heat shock protein 75 kDa, mitochondrial | 1.32 | 1.40 |
| Hepcidin | -5.01 | -13.21 |
| Heterogeneous nuclear ribonucleoprotein A/B | 1.45 | 1.66 |
| Heterogeneous nuclear ribonucleoprotein L |  | 2.88 |
| Heterogeneous nuclear ribonucleoprotein M | 2.93 |  |
| Heterogeneous nuclear ribonucleoprotein Q |  | 1.94 |
| Heterogeneous nuclear ribonucleoprotein U-like protein 1 | 1.46 |  |
| Histone H2A.V |  | 1.47 |
| Histone-arginine methyltransferase CARM1 | 1.47 |  |
| Histone-lysine N-methyltransferase EHMT2 | 2.01 | 2.06 |
| Histone-lysine N-methyltransferase SETD7 |  | 1.38 |
| Hormone-sensitive lipase-like protein | -2.26 |  |
| HRAS-like suppressor | -2.24 |  |
| Hsc70-interacting protein | 1.52 |  |
| Huntingtin-interacting protein K | 1.57 |  |
| Hyaluronidase-2 |  | -1.72 |
| Hydroxyacylglutathione hydrolase, mitochondrial | -1.47 | -1.35 |
| Hydroxymethylglutaryl-CoA lyase | -1.28 |  |
| Immediate early response gene 5-like protein |  | 1.86 |
| Importin-5 | 1.92 | 1.96 |
| Importin-7 | 1.53 |  |
| Importin-9 | 1.33 |  |
| Inositol monophosphatase 1 |  | 2.44 |
| Inositol monophosphatase 3 |  | 1.53 |
| **Gene Name** | **Fold-Change** | |
|  | **Hypo** | **Hyper** |
| ***Liver*** |  |  |
| Insulin-like growth factor 2 mRNA-binding protein 2 |  | -1.48 |
| Integrator complex subunit 8 | 2.50 | 2.64 |
| Integrin beta-1 | -3.30 |  |
| Inversin | -1.93 |  |
| Iron-sulfur cluster assembly enzyme ISCU, mitochondrial | -1.64 |  |
| Junctional adhesion molecule C |  | 2.24 |
| Kinesin-like protein KIF2A | 1.40 | 1.39 |
| Kinesin-like protein KIF2C | -4.46 |  |
| L antigen family member 3 | 1.81 |  |
| L-2-hydroxyglutarate dehydrogenase, mitochondrial | -4.89 | -5.95 |
| L-threonine 3-dehydrogenase, mitochondrial | -7.20 | -4.94 |
| Lamina-associated polypeptide 2, isoform beta | 1.55 | 1.70 |
| Lamina-associated polypeptide 2, isoforms beta/gamma |  | -1.99 |
| Laminin subunit alpha-2 | -1.70 | -1.49 |
| Leucine-rich PPR motif-containing protein, mitochondrial |  | 1.96 |
| Long-chain-fatty-acid--CoA ligase 1 | -1.72 |  |
| Long-chain-fatty-acid--CoA ligase ACSBG2 | -3.66 |  |
| Lymphocyte antigen 75 | -1.39 |  |
| Lymphoid-specific helicase |  | 2.02 |
| LYR motif-containing protein 2 | 1.46 | 1.42 |
| LYR motif-containing protein 7 | 1.59 | 1.74 |
| MAP/microtubule affinity-regulating kinase 3 | 1.38 | 1.54 |
| Matrix metalloproteinase-9 | -4.03 |  |
| Medium-chain specific acyl-CoA dehydrogenase, mitochondrial | -2.03 |  |
| Membrane magnesium transporter 1 | 1.34 | 1.28 |
| Membrane-associated progesterone receptor component 1 | -1.41 |  |
| Metallophosphoesterase 1 | 1.43 |  |
| Methylmalonate-semialdehyde dehydrogenase [acylating], mitochondrial | 1.83 |  |
| Methylmalonyl-CoA epimerase, mitochondrial | 1.51 | 1.58 |
| Microprocessor complex subunit DGCR8 |  | 1.91 |
| Mitochondrial import inner membrane translocase subunit Tim10 B | 2.07 |  |
| Mitochondrial import inner membrane translocase subunit tim16 | 1.51 |  |
| Mitochondrial import inner membrane translocase subunit Tim22 (JX975246) | 2.36 | 1.86 |
| Mitochondrial import inner membrane translocase subunit Tim8 A | 1.83 | 1.62 |
| Mitochondrial import receptor subunit TOM40 homolog | 2.55 | 2.78 |
| Mitochondrial intermembrane space import and assembly protein 40 | 1.83 |  |
| Mitochondrial ornithine transporter 1 |  | 2.10 |
| Mitogen-activated protein kinase 14A |  | -1.44 |
| Mitotic checkpoint serine/threonine-protein kinase BUB1 beta | -2.32 |  |
| mRNA export factor |  | 1.49 |
| mTERF domain-containing protein 2 |  | 1.93 |
| Multifunctional protein ADE2 | -1.93 |  |
| Multivesicular body subunit 12B |  | -1.99 |
| Musculoskeletal embryonic nuclear protein 1 | -1.87 |  |
| Myelin P2 protein | -1.89 |  |
| Myocyte-specific enhancer factor 2D homolog |  | -2.02 |
| N-acetylgalactosamine kinase | 1.41 |  |
| **Gene Name** | **Fold-Change** | |
|  | **Hypo** | **Hyper** |
| ***Liver*** |  |  |
| N-acetyltransferase 10 |  | -1.38 |
| N-acetyltransferase 14 |  | 2.03 |
| N-acetyltransferase ESCO2 | -1.33 | -1.66 |
| N-terminal EF-hand calcium-binding protein 1 | -6.73 |  |
| NAD kinase | -2.48 | -1.88 |
| NADH dehydrogenase [ubiquinone] 1 alpha subcomplex assembly factor 3 | 1.75 | 2.52 |
| NADH dehydrogenase [ubiquinone] 1 alpha subcomplex subunit 8 |  | 1.29 |
| NADH dehydrogenase [ubiquinone] 1 beta subcomplex subunit 5, mitochondrial |  | 1.42 |
| NADH-ubiquinone oxidoreductase 75 kDa subunit, mitochondrial | -1.42 |  |
| Nardilysin | 1.49 | 1.78 |
| Nascent polypeptide-associated complex subunit alpha | 1.65 |  |
| NEDD8-activating enzyme E1 catalytic subunit | 1.92 | 1.68 |
| Negative elongation factor D | -1.87 |  |
| Neural cell adhesion molecule L1-like protein |  | 2.23 |
| Nicalin |  | 1.55 |
| Nicotinamide phosphoribosyltransferase | 1.54 |  |
| Nitric oxide synthase-interacting protein | -1.26 |  |
| Nogo-B receptor |  | 2.66 |
| Non-specific lipid-transfer protein | -1.38 | -1.40 |
| Nuclear receptor coactivator 7 (140 kDa estrogen receptor-associated protein) (Estrogen nuclear receptor coactivator 1) | 2.60 |  |
| Nuclear ubiquitous casein and cyclin-dependent kinases substrate | 2.41 |  |
| Nucleolar GTP-binding protein 1 | 2.18 |  |
| Nucleolar protein 56 | 1.46 |  |
| Nucleolar RNA helicase 2 | 2.50 |  |
| Nucleoporin NDC1 | 1.59 | 1.77 |
| Nucleoside diphosphate kinase 7 |  | 1.32 |
| Oxysterol-binding protein-related protein 3 | -1.53 |  |
| Pantothenate kinase 2, mitochondrial | -1.45 |  |
| Pappalysin-1 | 4.44 |  |
| PC4 and SFRS1-interacting protein | 2.52 | 2.67 |
| Peptide-N(4)-(N-acetyl-beta-glucosaminyl)asparagine amidase | -1.34 |  |
| Peptidyl-prolyl cis-trans isomerase H |  | 1.59 |
| Peptidyl-prolyl cis-trans isomerase-like 3 | 1.95 | 1.90 |
| Periodic tryptophan protein 1 homolog |  | 1.79 |
| Peroxiredoxin-1 | -2.24 |  |
| Peroxiredoxin-2 | 1.48 |  |
| Peroxiredoxin-5, mitochondrial | -1.39 |  |
| Peroxisomal membrane protein PMP34 |  | 1.71 |
| Phosducin-like protein 3 | 1.55 |  |
| Phosphatase and actin regulator 4 |  | -1.65 |
| Phosphatidylinositide phosphatase SAC1-B | 1.50 |  |
| Phosphatidylinositol 3-kinase catalytic subunit type 3 | -1.47 |  |
| Phosphatidylinositol N-acetylglucosaminyltransferase subunit H | 1.95 |  |
| Phospholysine phosphohistidine inorganic pyrophosphate phosphatase | 1.58 |  |
| Pituitary tumor-transforming gene 1 protein-interacting protein | -1.84 | -1.78 |
| **Gene Name** | **Fold-Change** | |
|  | **Hypo** | **Hyper** |
| ***Liver*** |  |  |
| Plasma glutamate carboxypeptidase |  | 2.92 |
| Plasma kallikrein | -2.70 |  |
| Platelet endothelial cell adhesion molecule | -1.39 |  |
| Poly [ADP-ribose] polymerase 3 |  | 1.38 |
| Polyadenylate-binding protein 4 | 3.52 |  |
| Porphobilinogen deaminase | 3.18 | 3.37 |
| Pre-mRNA-processing factor 39 | 1.28 |  |
| Pre-mRNA-splicing factor 18 |  | -1.39 |
| Pre-rRNA-processing protein TSR2 homolog | 1.78 | 1.38 |
| Prefoldin subunit 2 | 1.67 |  |
| Probable 39S ribosomal protein L24, mitochondrial | 1.55 |  |
| Probable ATP-dependent RNA helicase DHX36 |  | 1.99 |
| Probable E3 ubiquitin-protein ligase TRIM8 | -1.40 | -1.41 |
| Probable G-protein coupled receptor 125 | -1.63 | -1.63 |
| Probable ribosome biogenesis protein RLP24 | 2.31 |  |
| Probable rRNA-processing protein EBP2 | 1.98 |  |
| Programmed cell death protein 5 |  | -1.72 |
| Propionyl-CoA carboxylase alpha chain, mitochondrial | -2.28 |  |
| Proteasome activator complex subunit 2 | -1.65 | -1.76 |
| Proteasome assembly chaperone 3 | 1.54 | 1.60 |
| Protein Churchill |  | 1.25 |
| Protein CLN8 | 1.44 |  |
| Protein cornichon homolog 4 |  | 1.87 |
| Protein FAM162B | -1.40 |  |
| Protein FAM189A2 | 1.91 |  |
| Protein FAM206A | 1.40 |  |
| Protein FAM36A | 1.53 | 1.74 |
| Protein FAM43A |  | 1.64 |
| Protein kinase C alpha type | -1.72 |  |
| Protein lgg-1 | -1.54 | -1.60 |
| Protein lin-37 homolog | 1.40 | 1.42 |
| Protein lin-52 homolog | 1.39 |  |
| Protein LLP homolog |  | 1.40 |
| Protein NLRC3 | 2.08 |  |
| Protein phosphatase 1 regulatory subunit 3C-B |  | -3.00 |
| Protein QIL1 |  | 1.33 |
| Protein RUFY3 |  | 1.39 |
| Protein saal1 | 2.36 | 2.16 |
| Protein transport protein Sec61 subunit alpha-like 1 | 1.87 |  |
| Protein VAC14 homolog |  | 1.37 |
| Protein YIF1B |  | 1.97 |
| Protein YIPF4 |  | -1.30 |
| Protein yippee-like 5 |  | -1.34 |
| Putative all-trans-retinol 13,14-reductase | -2.29 |  |
| Putative nuclease HARBI1 |  | 1.85 |
| Pyridoxal kinase | 3.10 |  |
| Pyridoxal phosphate phosphatase PHOSPHO2 | -2.03 | -1.96 |
| **Gene Name** | **Fold-Change** | |
|  | **Hypo** | **Hyper** |
| ***Liver*** |  |  |
| Queuine tRNA-ribosyltransferase | 2.10 | 1.82 |
| Rab proteins geranylgeranyltransferase component A 1 | -1.67 |  |
| Radial spoke head protein 9 homolog | 1.31 |  |
| RanBP-type and C3HC4-type zinc finger-containing protein 1 |  | -2.83 |
| Rap1 GTPase-activating protein 1 | 1.52 |  |
| Ras-related protein R-Ras2 |  | -1.48 |
| Ras-related protein Rab-11B |  | 1.57 |
| Ras-related protein Rab-2A | 1.29 |  |
| Ras-related protein Rab-9A | 1.34 |  |
| Ras-related protein Rap-1 |  | 1.48 |
| RB1-inducible coiled-coil protein 1 |  | -1.94 |
| RelA-associated inhibitor | -2.32 |  |
| Renin receptor |  | 1.30 |
| Retinoic acid receptor RXR-beta-A |  | -1.61 |
| Rhomboid-related protein 2 | 1.45 |  |
| Ribonuclease T2 | -1.25 |  |
| Ribosome biogenesis protein NSA2 homolog | 2.12 |  |
| RILP-like protein 1 |  | -1.94 |
| RING finger protein 113A |  | 2.46 |
| RING finger protein 185 | -1.47 |  |
| RNA polymerase II-associated protein 3 | 1.30 |  |
| RNA-binding protein PNO1 | 2.06 |  |
| RRP12-like protein |  | 1.94 |
| SAC3 domain-containing protein 1 |  | 1.96 |
| SAM and SH3 domain-containing protein 3 | -3.32 |  |
| Selenoprotein H | -1.68 |  |
| Septin-2 |  | 1.27 |
| Serine incorporator 3 | -1.40 |  |
| Serine-threonine kinase receptor-associated protein |  | 1.35 |
| Serine/arginine-rich splicing factor 2 |  | 1.36 |
| Serine/arginine-rich splicing factor 7 | 1.59 |  |
| Serine/threonine-protein kinase 35 | 1.79 |  |
| Serine/threonine-protein kinase TBK1 | -1.40 |  |
| Serine/threonine-protein kinase/endoribonuclease IRE2 |  | 1.34 |
| Serine/threonine-protein phosphatase 2A 55 kDa regulatory subunit B alpha isoform | 1.67 |  |
| Serine/threonine-protein phosphatase 2A 55 kDa regulatory subunit B delta isoform | -1.64 | -1.66 |
| SH3 domain and tetratricopeptide repeats-containing protein 1 |  | -1.68 |
| Shugoshin-like 1 | -3.34 |  |
| Sialic acid synthase |  | 1.37 |
| Sialic acid-binding Ig-like lectin 15 |  | -2.51 |
| Signal recognition particle 68 kDa protein | 1.86 |  |
| Signal recognition particle 9 kDa protein |  | 1.36 |
| Signal transducer and activator of transcription 6 |  | -1.82 |
| SLIT and NTRK-like protein 6 | 3.27 |  |
| Small EDRK-rich factor 2 | 1.33 |  |
| **Gene Name** | **Fold-Change** | |
|  | **Hypo** | **Hyper** |
| ***Liver*** |  |  |
| Small nuclear ribonucleoprotein E | 1.71 |  |
| Small nuclear ribonucleoprotein F | 1.72 | 1.85 |
| Small nuclear ribonucleoprotein Sm D2 |  | 1.92 |
| Small ubiquitin-related modifier 3 | 1.50 | 1.59 |
| Snurportin-1 | -1.38 |  |
| Solute carrier family 22 member 4 | 1.39 |  |
| Solute carrier family 25 member 38-B | -5.24 | -4.05 |
| Solute carrier family 25 member 39 |  | -1.81 |
| Solute carrier family 25 member 42 |  | -1.80 |
| Solute carrier family 25 member 43 |  | -1.59 |
| Solute carrier family 25 member 48 | -4.55 | -6.32 |
| Solute carrier family 41 member 2 |  | 2.64 |
| SOSS complex subunit C | 1.47 | 1.60 |
| Sphingomyelin phosphodiesterase 4 |  | 1.65 |
| Spindlin-3 |  | 1.55 |
| Splicing factor, proline- and glutamine-rich |  | -1.44 |
| Staphylococcal nuclease domain-containing protein 1 | 1.46 |  |
| Striatin |  | -1.95 |
| Succinate dehydrogenase [ubiquinone] cytochrome b small subunit B, mitochondrial (KC217618) | 1.34 | 1.67 |
| Succinate dehydrogenase [ubiquinone] flavoprotein subunit, mitochondrial |  | 1.23 |
| Succinate dehydrogenase [ubiquinone] iron-sulfur subunit, mitochondrial |  | 2.23 |
| Suppressor of IKBKE 1 | -1.61 | -1.55 |
| SWI/SNF-related matrix-associated actin-dependent regulator of chromatin subfamily B member 1 |  | 1.51 |
| Syntaxin-binding protein 6 | -1.51 | -1.58 |
| T-complex protein 1 subunit delta | 1.80 |  |
| T-complex protein 1 subunit epsilon | 1.93 |  |
| T-complex protein 1 subunit eta | 1.94 |  |
| Tafazzin | 1.35 | 1.34 |
| TAR DNA-binding protein 43 | 1.48 | 1.41 |
| Tax1-binding protein 1 homolog B | -1.55 | -1.66 |
| TBC1 domain family member 7 | 1.61 |  |
| Telomerase protein component 1 | -1.77 |  |
| Tetraspanin-13 |  | 1.30 |
| Tetraspanin-5 |  | -1.95 |
| THAP domain-containing protein 2 |  | 1.58 |
| Thioredoxin-like protein 4B | 1.62 | 1.54 |
| Thioredoxin-related transmembrane protein 2-B | 1.64 | 2.58 |
| THO complex subunit 5 homolog |  | 1.29 |
| TM2 domain-containing protein 1 |  | 1.25 |
| TNFAIP3-interacting protein 1 | -1.37 |  |
| Trafficking protein particle complex subunit 8 | -3.98 | -2.81 |
| Transcription factor BTF3 | 1.80 |  |
| Transcription factor HES-5 |  | 2.76 |
| Transcription factor p65 |  | -2.83 |
| Transcription initiation factor TFIID subunit 2 |  | 1.35 |
| **Gene Name** | **Fold-Change** | |
|  | **Hypo** | **Hyper** |
| ***Liver*** |  |  |
| Transcription termination factor, mitochondrial |  | 2.44 |
| Transcriptional regulator ERG | 1.46 | 1.42 |
| Transcriptional repressor CTCF |  | 1.40 |
| Transcriptional repressor p66-alpha |  | -1.64 |
| Transcriptional repressor protein YY1 |  | -2.12 |
| Transketolase-like protein 2 | -1.34 |  |
| Translationally-controlled tumor protein homolog | 1.49 |  |
| Translocon-associated protein subunit alpha | 1.57 |  |
| Translocon-associated protein subunit beta | 1.28 |  |
| Transmembrane 9 superfamily member 3 |  | 1.40 |
| Transmembrane channel-like protein 6 | 1.68 |  |
| Transmembrane emp24 domain-containing protein 7 |  | 1.28 |
| Transmembrane protein 184A | -2.13 |  |
| Transmembrane protein 223 | 1.73 | 1.96 |
| Transmembrane protein 33 | 2.50 |  |
| Transmembrane protein 41A-A | 1.92 |  |
| Trifunctional enzyme subunit alpha, mitochondrial |  | 2.12 |
| Tropomyosin beta chain | 2.70 | 2.31 |
| Tubulin alpha chain | -1.77 |  |
| Tubulin delta chain | 1.55 |  |
| Twinfilin-1 | 1.45 | 1.44 |
| Tyrosine-protein phosphatase non-receptor type 12 | -1.95 | -2.00 |
| Tyrosyl-DNA phosphodiesterase 2 | 1.65 |  |
| Tyrosyl-tRNA synthetase, cytoplasmic |  | 2.61 |
| U5 small nuclear ribonucleoprotein 200 kDa helicase |  | 1.50 |
| Ubiquitin fusion degradation protein 1 homolog | -1.45 | -1.39 |
| Ubiquitin-conjugating enzyme E2 N (KM522797) | 1.44 | 1.39 |
| Ubiquitin-conjugating enzyme E2 variant 1 |  | 1.61 |
| Ubiquitin-like protein FUBI | 1.37 |  |
| Ubiquitin-related modifier 1 homolog |  | 1.99 |
| UDP-glucuronosyltransferase 2A3 |  | 1.57 |
| UDP-xylose and UDP-N-acetylglucosamine transporter | 2.61 |  |
| UMP-CMP kinase 2, mitochondrial | 4.38 | 3.52 |
| Uncharacterized protein C11orf51 homolog | 2.73 | 2.50 |
| Uncharacterized protein C15orf57 |  | 1.21 |
| Uncharacterized protein C16orf61 homolog | 1.29 |  |
| Uncharacterized protein C19orf52 | 1.34 | 1.38 |
| Uncharacterized protein C7orf44 |  | 3.11 |
| Uncharacterized protein KIAA1211 | -5.43 | -4.56 |
| Uncharacterized protein KIAA1310 homolog |  | -1.65 |
| UPF0160 protein MYG1, mitochondrial |  | 1.55 |
| UPF0368 protein Cxorf26 | 1.78 | 1.76 |
| UPF0369 protein C6orf57 | 2.14 | 1.93 |
| UPF0545 protein C22orf39 homolog | 1.40 |  |
| UPF0609 protein C4orf27 homolog | 1.59 |  |
| UPF0683 protein C7orf47 homolog | -1.66 |  |
| Urokinase plasminogen activator surface receptor | -2.82 |  |
| **Gene Name** | **Fold-Change** | |
|  | **Hypo** | **Hyper** |
| ***Liver*** |  |  |
| V-type proton ATPase subunit E 1 | 1.84 | 1.68 |
| Vacuolar fusion protein CCZ1 homolog |  | -1.79 |
| Vacuolar protein sorting-associated protein 37A | -1.17 | -1.31 |
| Vacuolar protein sorting-associated protein VTA1 homolog | 1.39 |  |
| Vinculin |  | 1.55 |
| Vitamin K-dependent protein S | -1.61 |  |
| Voltage-dependent anion-selective channel protein 2 |  | 1.77 |
| WW domain-binding protein 11 |  | 1.60 |
| WW domain-containing adapter protein with coiled-coil | -1.37 | -1.37 |
| Zinc finger and BTB domain-containing protein 8A | -2.75 | -2.67 |
| Zinc finger matrin-type protein 5 | 1.41 |  |
| Zinc finger MYM-type protein 5 | 1.38 |  |
| Zinc finger protein 10 |  | 1.52 |
| Zinc finger protein 276 |  | -1.45 |
| Zinc finger protein 36, C3H1 type-like 2 | 1.75 |  |
| Zinc finger protein 593 | 1.50 |  |
| Zinc finger protein 598 | 1.63 | 1.40 |
| Zinc finger protein 624 |  | -1.63 |
| Zinc finger protein 644 | 1.22 | 1.20 |
| Zinc finger protein 721 |  | 1.67 |
| Zinc transporter ZIP13 | 1.79 | 1.80 |
| ***Gills*** |  |  |
| 60S ribosomal protein L38 |  | 1.38 |
| Actin-binding LIM protein 2 | 2.31 |  |
| Adiponectin receptor protein 1 | -1.26 |  |
| ADP,ATP carrier protein 1 | 1.52 |  |
| Alanine--glyoxylate aminotransferase 2-like 1 | -2.99 | -4.44 |
| Alkaline phosphatase |  | 1.42 |
| Alpha-enolase |  | 2.08 |
| Alpha-glucosidase 2 | -4.90 |  |
| Alpha-tectorin | -2.26 |  |
| Annexin A1 |  | 1.92 |
| Annexin A2 |  | 4.90 |
| Anterior gradient protein 2 homolog |  | 2.65 |
| Apolipoprotein A-I | 7.73 |  |
| Apoptosis regulator BAX | -1.32 |  |
| Arachidonate 12-lipoxygenase, 12R-type |  | 7.38 |
| ATP synthase-coupling factor 6, mitochondrial | -2.28 |  |
| BAG family molecular chaperone regulator 2 |  | 1.53 |
| Basigin | 1.28 | 1.33 |
| Beclin-1 | -1.24 |  |
| Beta-microseminoprotein |  | 6.32 |
| Calcium-binding protein p22 | 1.99 |  |
| Calpain-9 | -2.04 |  |
| Carbonic anhydrase 6 (EC 4.2.1.1) (Carbonate dehydratase VI) (Carbonic anhydrase VI) (CA-VI) (Salivary carbonic anhydrase) (Secreted carbonic anhydrase) | 4.54 |  |
| **Gene Name** | **Fold-Change** | |
|  | **Hypo** | **Hyper** |
| ***Gills*** |  |  |
| Carbonyl reductase [NADPH] 1 |  | 1.65 |
| Carboxymethylenebutenolidase homolog | 1.56 |  |
| CC chemokine CK7 | 5.16 | 4.61 |
| CDGSH iron-sulfur domain-containing protein 1 |  | 1.35 |
| Cholesterol 7-alpha-monooxygenase |  | 2.68 |
| Coatomer subunit alpha | 1.64 |  |
| Coiled-coil-helix-coiled-coil-helix domain-containing protein 7 | -1.30 |  |
| Complement factor I | -2.69 | -2.83 |
| Cystatin-A1 | -4.94 | -4.44 |
| Cysteine-rich protein 2 |  | 3.11 |
| Cytidine deaminase |  | 14.24 |
| Cytochrome b-c1 complex subunit 1, mitochondrial |  | 1.48 |
| Cytochrome b5 | 1.73 |  |
| Cytochrome c oxidase subunit 5A, mitochondrial |  | 1.68 |
| Cytochrome c oxidase subunit 8B, mitochondrial (KC217648) |  | 1.85 |
| Cytochrome P450 1A1 | 7.57 |  |
| Cytochrome P450 1B1 |  | 3.43 |
| Cytosolic sulfotransferase 2 | 1.91 |  |
| Dynein light chain Tctex-type 1 | -1.60 |  |
| Eosinophil peroxidase |  | 3.42 |
| Epigen |  | 2.69 |
| Eukaryotic translation initiation factor 4 gamma 1 | 1.59 |  |
| Fructose-bisphosphate aldolase C-B |  | 1.76 |
| FXYD domain-containing ion transport regulator 5 |  | -1.44 |
| Galectin-9 |  | -1.41 |
| Gamma-aminobutyric acid receptor-associated protein-like 2 |  | 1.45 |
| Gastrin | 8.47 |  |
| GDNF-inducible zinc finger protein 1 | -1.55 |  |
| General transcription factor IIF subunit 1 | 5.16 |  |
| Glia maturation factor gamma | -1.27 |  |
| Glucosamine 6-phosphate N-acetyltransferase |  | 1.31 |
| Glutamate--cysteine ligase catalytic subunit | 1.74 |  |
| Glutathione S-transferase A | 1.81 | 2.47 |
| Glyceraldehyde 3-phosphate dehydrogenase, testis-specific |  | 1.50 |
| Glycogen phosphorylase, brain form | -1.67 |  |
| Glycogen phosphorylase, brain form (Fragment) | 2.50 |  |
| Glycosylphosphatidylinositol anchor attachment 1 protein | 1.36 |  |
| Glyoxalase domain-containing protein 4 | 1.56 |  |
| H-2 class I histocompatibility antigen, K-K alpha chain | -233.08 |  |
| H-2 class I histocompatibility antigen, Q8 alpha chain | 14.80 |  |
| Heat shock protein beta-8 |  | 1.71 |
| Heat shock protein HSP 90-alpha | 2.29 |  |
| Hepatocyte growth factor-like protein |  | 2.97 |
| Hepcidin |  | -1.33 |
| IgGFc-binding protein |  | 3.71 |
| Inhibitor of growth protein 5 | -1.28 |  |
| Integrin beta-2 | -1.31 |  |
| **Gene Name** | **Fold-Change** | |
|  | **Hypo** | **Hyper** |
| ***Gills*** |  |  |
| Interleukin-6 receptor subunit alpha (JX976616) | 1.48 |  |
| Junction plakoglobin | 1.50 | 1.48 |
| Keratin, type I cytoskeletal 13 | 4.97 |  |
| Keratin, type I cytoskeletal 19 |  | 2.22 |
| Ketohexokinase |  | 2.68 |
| Lactosylceramide alpha-2,3-sialyltransferase |  | -1.54 |
| Macrophage-expressed gene 1 protein | -2.19 |  |
| Malate dehydrogenase, cytoplasmic |  | 1.75 |
| Matrilin-2 | 2.92 |  |
| Minor histocompatibility protein HA-1 | -1.33 |  |
| Mitogen-activated protein kinase 6 |  | 1.48 |
| MKRN2 opposite strand protein |  | 2.96 |
| Multiple inositol polyphosphate phosphatase 1 | 2.08 |  |
| Myelin-oligodendrocyte glycoprotein |  | -2.18 |
| Myosin heavy chain, fast skeletal muscle | 6.06 |  |
| NADH-cytochrome b5 reductase |  | 2.33 |
| Neuroblast differentiation-associated protein AHNAK |  | 1.51 |
| Oligoribonuclease, mitochondrial |  | 1.43 |
| Pancreatic secretory trypsin inhibitor |  | 2.50 |
| Peroxisome proliferator-activated receptor gamma | 1.49 |  |
| PEST proteolytic signal-containing nuclear protein | -1.20 |  |
| Phosphatidylinositide phosphatase SAC1-B |  | 1.46 |
| Phosphoenolpyruvate carboxykinase, cytosolic [GTP] |  | 6.89 |
| Phosphoinositide 3-kinase adapter protein 1 |  | -1.41 |
| Pleckstrin homology domain-containing family B member 2 | -2.29 |  |
| Potassium channel subfamily K member 5 | 2.83 |  |
| PREDICTED: layilin-like [Maylandia zebra] | 1.85 |  |
| Protein ETHE1, mitochondrial | 1.52 |  |
| Protein FAM36A |  | 1.43 |
| Protein FAM8A1 | -1.45 | -1.39 |
| Protein NipSnap homolog 3A | -1.31 |  |
| Protein S100-A1 | -5.08 |  |
| Protein S100-A16 |  | 1.97 |
| Protein S100-A6 |  | 2.26 |
| Putative adenosylhomocysteinase 3 |  | 3.56 |
| Putative ribosomal RNA methyltransferase 1 | -2.96 |  |
| Rab11 family-interacting protein 1 | 1.41 |  |
| Ras GTPase-activating protein 3 | 1.45 |  |
| Regulation of nuclear pre-mRNA domain-containing protein 2 |  | -3.89 |
| Rhombotin-1 | -2.35 |  |
| RING finger protein 114 | -1.26 |  |
| S-adenosylmethionine synthase isoform type-2 | 1.62 |  |
| SAM and SH3 domain-containing protein 3 |  | -3.31 |
| Selenoprotein Pa | -1.47 |  |
| Sentan | 2.48 | 2.73 |
| Short coiled-coil protein | 1.70 |  |
| Sodium/potassium-transporting ATPase subunit alpha-1 |  | 1.70 |
| **Gene Name** | **Fold-Change** | |
|  | **Hypo** | **Hyper** |
| ***Gills*** |  |  |
| Sortilin | 2.83 |  |
| Succinate dehydrogenase [ubiquinone] flavoprotein subunit, mitochondrial |  | 1.40 |
| Succinyl-CoA ligase [GDP-forming] subunit beta, mitochondrial (Fragment) |  | 1.51 |
| T-cell activation Rho GTPase-activating protein | -1.37 | -1.29 |
| Transmembrane glycoprotein NMB |  | 9.55 |
| Tuftelin | -1.60 |  |
| Ubiquitin carboxyl-terminal hydrolase CYLD |  | -1.57 |
| UDP-glucuronosyltransferase (Fragment) | 2.02 |  |
| UDP-glucuronosyltransferase 2A3 |  | 1.52 |
| UPF0764 protein C16orf89 homolog | -2.75 |  |
| Uricase |  | 2.22 |
| Voltage-dependent anion-selective channel protein 2 | 1.51 |  |
| Zinc finger Ran-binding domain-containing protein 3 |  | -2.29 |
| Zona pellucida sperm-binding protein 2 | 1.62 | 1.64 |
| ***Hypothalamus*** |  |  |
| 60S ribosomal protein L10a | 2.77 |  |
| 60S ribosomal protein L13a (Fragment) |  | -2.65 |
| A disintegrin and metalloproteinase with thrombospondin motifs 17 | -3.60 | -3.46 |
| A-kinase anchor protein 11 | -1.67 |  |
| Actin | 3.59 |  |
| Acyl-CoA synthetase family member 2, mitochondrial | 2.30 |  |
| Acyl-CoA-binding domain-containing protein 5 | -4.85 | -4.03 |
| Aldehyde dehydrogenase, mitochondrial | 1.75 |  |
| Alkaline phosphatase | 1.48 |  |
| Allantoicase | -3.24 |  |
| Amyloid beta A4 precursor protein-binding family A member 1 |  | -3.27 |
| Annexin A2 | 2.84 |  |
| Apoptosis-associated speck-like protein containing a CARD | 2.25 |  |
| Arf-GAP with Rho-GAP domain, ANK repeat and PH domain-containing protein 1 |  | -7.26 |
| Band 4.1-like protein 5 | 1.76 |  |
| Beta-ureidopropionase | 1.79 |  |
| C-C chemokine receptor type 7 | -2.13 | -2.31 |
| C-type lectin domain family 4 member K | -1.63 |  |
| Cadherin-1 (KF861995) |  | -1.75 |
| Calcitonin gene-related peptide type 1 receptor | 1.96 |  |
| Calpain 3 (KM522785) |  | -3.33 |
| Calpain small subunit 1 | 2.08 | 1.74 |
| Carboxylesterase 5A | 2.93 |  |
| Caspase recruitment domain-containing protein 9 | -2.71 | -2.62 |
| Charged multivesicular body protein 5 | 2.39 | 2.31 |
| Chloride intracellular channel protein 2 | 1.79 |  |
| Chymotrypsin-like elastase family member 2A | 4.12 | 3.03 |
| Claudin-10 | -3.76 |  |
| Collagen alpha-1(I) chain | 5.36 |  |
| Collagen alpha-1(VIII) chain | 2.17 |  |
| Collagen alpha-1(XI) chain | 3.28 |  |
| **Gene Name** | **Fold-Change** | |
|  | **Hypo** | **Hyper** |
| ***Hypothalamus*** |  |  |
| Collagen alpha-2(I) chain | 3.96 |  |
| Collagen alpha-2(V) chain | 2.36 |  |
| Collagen alpha-3(VI) chain | 2.49 |  |
| COMM domain-containing protein 2 | 2.75 |  |
| Complement component C7 | 2.84 |  |
| Complement factor I | -3.71 |  |
| Cryptochrome-1 |  | 1.74 |
| Cytochrome P450 1B1 | 3.21 |  |
| Cytochrome P450 2J2 | 2.77 |  |
| Cytokine-like protein 1 | 2.46 |  |
| Cytosolic 5'-nucleotidase 1A |  | -1.44 |
| Cytosolic sulfotransferase 2 | 4.69 |  |
| DENN domain-containing protein 3 | -2.78 |  |
| Desmin (KM522780) | -6.51 | -10.06 |
| Dipeptidyl peptidase 4 | 3.18 |  |
| Dual specificity mitogen-activated protein kinase kinase 2 | 2.86 | 6.40 |
| E-selectin | 2.74 |  |
| Engulfment and cell motility protein 2 |  | -4.80 |
| Ephrin type-A receptor 2 | 1.56 |  |
| Eukaryotic initiation factor 4A-II |  | 1.55 |
| Eukaryotic translation initiation factor 3 subunit F |  | -1.64 |
| Extracellular superoxide dismutase [Cu-Zn] | 1.66 |  |
| F-box only protein 6 | 2.03 |  |
| Far upstream element-binding protein 1 |  | -1.90 |
| Fermitin family homolog 3 |  | -2.64 |
| Fibulin-5 |  | 2.21 |
| Gamma-glutamyl hydrolase | 2.54 |  |
| GC-rich sequence DNA-binding factor 1 |  | -1.45 |
| Glutathione S-transferase A |  | 1.82 |
| GRAM domain-containing protein 2 | 2.92 |  |
| Growth factor receptor-bound protein 14 | -1.76 | -2.10 |
| Heat-stable enterotoxin receptor |  | -2.01 |
| Hemoglobin subunit beta | 2.64 |  |
| Hephaestin-like protein 1 | -3.02 |  |
| Homogentisate 1,2-dioxygenase | 2.93 | 2.39 |
| IGF-like family receptor 1 | 2.23 |  |
| Indoleamine 2,3-dioxygenase 2 |  | -2.18 |
| Interleukin-1 receptor-like 2 | 2.80 |  |
| Junctional adhesion molecule A (KF861997) |  | -1.87 |
| Keratin, type II cytoskeletal |  | -3.32 |
| Keratinocyte-associated protein 3 | -2.12 | -2.28 |
| Kielin/chordin-like protein | 2.21 |  |
| Lamin-A | -2.38 |  |
| Laminin subunit beta-1 | 2.23 |  |
| Latexin | 1.63 |  |
| Lipoprotein lipase | 1.57 |  |
| Lysyl oxidase homolog 3 |  | 3.05 |
| **Gene Name** | **Fold-Change** | |
|  | **Hypo** | **Hyper** |
| ***Hypothalamus*** |  |  |
| Macrophage mannose receptor 1 |  | -2.61 |
| Macrophage-expressed gene 1 protein | -2.49 | -2.40 |
| Major facilitator superfamily domain-containing protein 1 | -2.15 |  |
| Mannan-binding lectin serine protease 2 | 1.91 |  |
| Mediator of RNA polymerase II transcription subunit 22 | 1.73 |  |
| Metaxin-2 |  | 1.69 |
| Microsomal triglyceride transfer protein large subunit | 2.80 |  |
| Mps one binder kinase activator-like 1B | 3.32 |  |
| Murinoglobulin-2 | -2.68 | -2.51 |
| Myeloma-overexpressed gene 2 protein |  | 1.30 |
| Myocilin | 2.47 |  |
| N-acetyltransferase ESCO2 | 4.87 |  |
| NACHT, LRR and PYD domains-containing protein 1 |  | -3.14 |
| NADH-ubiquinone oxidoreductase chain 1 (KC217558) |  | -2.17 |
| Neuroblast differentiation-associated protein AHNAK | 1.62 |  |
| Neutrophil elastase | 5.70 |  |
| Non-muscle caldesmon | 1.64 |  |
| P2Y purinoceptor 1 | 2.51 |  |
| Periostin | 3.59 |  |
| Peroxisomal proliferator-activated receptor A-interacting complex 285 kDa protein |  | -1.54 |
| Phosphoenolpyruvate carboxykinase, cytosolic [GTP] |  | 7.56 |
| Phosphorylase b kinase regulatory subunit alpha, liver isoform |  | 2.36 |
| Prefoldin subunit 3 | 2.67 |  |
| Pro-cathepsin H | 1.76 |  |
| Probable ATP-dependent RNA helicase DDX5 |  | -1.44 |
| Probable ribonuclease ZC3H12B |  | -1.36 |
| Procollagen C-endopeptidase enhancer 2 | 2.14 |  |
| Protein FAM176B |  | 2.64 |
| Protein FAM57A |  | -1.52 |
| Protein HIDE1 | 2.02 |  |
| Protein ITFG3 | 1.33 |  |
| Protein odd-skipped-related 1 | 2.38 |  |
| Protein RUFY3 | 1.45 | 1.37 |
| Putative phospholipase B-like 2 | -3.08 | -3.22 |
| Ras association domain-containing protein 1 |  | 1.94 |
| Ras GTPase-activating protein 1 |  | -2.07 |
| Ras-related protein Rab-18A |  | -1.52 |
| Ras-related protein Rab-6B |  | -1.98 |
| Receptor activity-modifying protein 2 | 2.28 |  |
| Retinoid-binding protein 7 | 12.09 |  |
| Rhomboid domain-containing protein 1 | -2.87 | -2.72 |
| Ribonuclease inhibitor |  | -1.79 |
| Selenium-binding protein 1 | 1.79 |  |
| Sentrin-specific protease 1 | 1.25 |  |
| Septin-7-like |  | -6.31 |
| Serine incorporator 1 | 1.49 |  |
| **Gene Name** | **Fold-Change** | |
|  | **Hypo** | **Hyper** |
| ***Hypothalamus*** |  |  |
| Serine--pyruvate aminotransferase, mitochondrial | -2.29 |  |
| Solute carrier family 22 member 6-A | 2.06 |  |
| Solute carrier family 25 member 34 |  | -2.28 |
| Solute carrier family 26 member 6 | 3.71 |  |
| Spartin |  | -1.64 |
| SPRY domain-containing SOCS box protein 4 |  | -2.48 |
| Stathmin | 3.12 |  |
| Steroidogenic acute regulatory protein, mitochondrial | 2.62 |  |
| Supervillin |  | -1.77 |
| Tax1-binding protein 1 homolog B |  | -1.84 |
| Terminal uridylyltransferase 7 |  | -1.61 |
| Tetraspanin-14 | 1.19 |  |
| Tetraspanin-17 |  | -1.30 |
| Tetraspanin-4 | 1.64 |  |
| Tetratricopeptide repeat protein 31 | 1.25 |  |
| Thrombomodulin | 1.52 |  |
| Tissue factor pathway inhibitor | 2.32 | 2.08 |
| TLC domain-containing protein 2 | 1.61 |  |
| Transferrin receptor protein 1 | 2.14 |  |
| Transforming growth factor beta-1 |  | -1.99 |
| Transmembrane protein 100 | -2.57 |  |
| Tropomyosin alpha-1 chain |  | -1.87 |
| Troponin I, cardiac muscle | -2.49 |  |
| Tubulin alpha-1B chain | -2.05 |  |
| Ubiquitin carboxyl-terminal hydrolase 11 |  | -1.72 |
| Ubiquitin carboxyl-terminal hydrolase CYLD |  | -1.77 |
| UBX domain-containing protein 4 |  | -1.42 |
| UBX domain-containing protein 7 |  | -1.94 |
| UDP-GlcNAc:betaGalbeta-1,3-N-acetylglucosaminyltransferase 3 |  | -1.94 |
| UDP-glucuronosyltransferase 1-9 |  | -1.76 |
| Uncharacterized protein C15orf57 |  | -1.83 |
| UPF0687 protein C20orf27 |  | -1.56 |
| Uricase | -2.23 |  |
| Vitamin K epoxide reductase complex subunit 1-like protein 1 | 2.75 | 1.79 |
| Xin actin-binding repeat-containing protein 2 |  | -1.54 |
| Zinc finger protein RFP | 2.47 |  |

**S1 File Table B. Differentially expressed genes mapped in overlapping pathway charts of liver after hypo- (Hypo) and hyper- (Hyper) challenge referred to SW acclimated fish (control, 38 ppt).** The clone code for each gene in the Nutrigroup database (www.nutrigroup-iats.org/seabreamdb) is indicated. Up- (🡹) and down- (🡻) regulation, as well as no variations (=) in all genes after each salinity transfer is also specified.

| ***Clone*** | ***Gene description*** | ***Hypo*** | ***Hyper*** | ***Canonical pathways*** | | | | | | | | | | | | | |
| --- | --- | --- | --- | --- | --- | --- | --- | --- | --- | --- | --- | --- | --- | --- | --- | --- | --- |
| C2_4023_u | 10 kDa heat shock protein, mitochondrial (JX975224) | 🡹 | 🡹 | 10 | 11 |  |  |  |  |  |  |  |  |  |  |  |  |
| [C2_9582](http://nutrigroup-iats.org/seabreamdb/blastResultWww.php#16779) | 2-oxoglutarate dehydrogenase, mitochondrial | 🡹 | = | 2 |  |  |  |  |  |  |  |  |  |  |  |  |  |
| C2_12280 | 40S ribosomal protein S11 | 🡹 | 🡹 | 3 | 4 | 5 |  |  |  |  |  |  |  |  |  |  |  |
| C2_583 | 40S ribosomal protein S12 | 🡹 | = | 3 | 4 | 5 |  |  |  |  |  |  |  |  |  |  |  |
| C2_7337 | 40S ribosomal protein S16 | 🡹 | = | 3 | 4 | 5 |  |  |  |  |  |  |  |  |  |  |  |
| C2_684 | 40S ribosomal protein S2 | 🡹 | = | 3 | 4 | 5 |  |  |  |  |  |  |  |  |  |  |  |
| C2_1271 | 40S ribosomal protein S24 | 🡹 | = | 3 | 4 | 5 |  |  |  |  |  |  |  |  |  |  |  |
| C2_8009 | 5'-AMP-activated protein kinase subunit beta-1 | = | 🡻 | 4 |  |  |  |  |  |  |  |  |  |  |  |  |  |
| C2_5222 | 60 kDa heat shock protein, mitochondrial | = | 🡹 | 10 | 11 |  |  |  |  |  |  |  |  |  |  |  |  |
| C2_282 | 60S ribosomal protein L10a | 🡹 | = | 3 |  |  |  |  |  |  |  |  |  |  |  |  |  |
| C2_441 | 60S ribosomal protein L13a (Fragment) | 🡻 | 🡻 | 3 |  |  |  |  |  |  |  |  |  |  |  |  |  |
| C2_98 | 60S ribosomal protein L7a | 🡹 | = | 3 |  |  |  |  |  |  |  |  |  |  |  |  |  |
| C2_156 | 60S ribosomal protein L9 | 🡹 | 🡹 | 3 |  |  |  |  |  |  |  |  |  |  |  |  |  |
| C2_42_u | 90kDa heat shock protein beta (KM522803) | 🡹 | = | 10 | 11 | 15 | 16 |  |  |  |  |  |  |  |  |  |  |
| C2_33733 | Actin-related protein 2/3 complex subunit 4 | = | 🡹 | 6 | 7 | 8 | 14 | 18 |  |  |  |  |  |  |  |  |  |
| C2_503 | Actin-related protein 2/3 complex subunit 5 | 🡹 | 🡹 | 6 | 7 | 8 | 14 | 18 |  |  |  |  |  |  |  |  |  |
| C2_1771 | Actin-related protein 3 | = | 🡹 | 6 | 7 | 8 | 14 | 18 |  |  |  |  |  |  |  |  |  |
| C2_1001 | ADP-ribosylation factor 1 | = | 🡹 | 13 | 14 |  |  |  |  |  |  |  |  |  |  |  |  |
| C2_22132 | Aldehyde dehydrogenase family 3 member B1 | 🡻 | 🡻 | 15 | 16 |  |  |  |  |  |  |  |  |  |  |  |  |
| C2_2673 | ATP synthase mitochondrial F1 complex assembly factor 2 | 🡹 | 🡹 | 1 | 2 |  |  |  |  |  |  |  |  |  |  |  |  |
| C2_49807 | ATP synthase subunit d, mitochondrial | 🡹 | = | 1 | 2 |  |  |  |  |  |  |  |  |  |  |  |  |
| ***Clone*** | ***Gene description*** | ***Hypo*** | ***Hyper*** | ***Canonical pathways*** | | | | | | | | | | | | | |
| C2_13281 | C-X-C chemokine receptor type 7 | = | 🡻 | 6 |  |  |  |  |  |  |  |  |  |  |  |  |  |
| C2_3963 | Calpain-1 catalytic subunit | 🡹 | = | 14 |  |  |  |  |  |  |  |  |  |  |  |  |  |
| C2_14726 | Carbonyl reductase [NADPH] 1 | 🡻 | = | 9 |  |  |  |  |  |  |  |  |  |  |  |  |  |
| C2_997_u | Catalase (CPT1A) (JQ308823) | 🡻 | 🡻 | 2 | 9 | 16 |  |  |  |  |  |  |  |  |  |  |  |
| C2_137 | Cathepsin D | 🡻 | = | 15 |  |  |  |  |  |  |  |  |  |  |  |  |  |
| C2_16199 | Cullin-1 | = | 🡻 | 11 |  |  |  |  |  |  |  |  |  |  |  |  |  |
| C2_8602 | Cyclin-dependent kinase 2 | = | 🡹 | 15 | 17 |  |  |  |  |  |  |  |  |  |  |  |  |
| C2_1152 | Cytochrome b-c1 complex subunit 1, mitochondrial | 🡹 | = | 1 | 2 |  |  |  |  |  |  |  |  |  |  |  |  |
| C2_2080 | Cytochrome b-c1 complex subunit 10 | 🡹 | = | 1 | 2 |  |  |  |  |  |  |  |  |  |  |  |  |
| C2_633 | Cytochrome b-c1 complex subunit 2, mitochondrial | = | 🡹 | 1 | 2 |  |  |  |  |  |  |  |  |  |  |  |  |
| C2_507 | Cytochrome b-c1 complex subunit Rieske, mitochondrial | = | 🡹 | 1 | 2 |  |  |  |  |  |  |  |  |  |  |  |  |
| C2_33419 | Cytochrome c oxidase assembly protein COX11, mitochondrial | = | 🡹 | 1 | 2 |  |  |  |  |  |  |  |  |  |  |  |  |
| C2_238 | Cytochrome c oxidase subunit 5A, mitochondrial | 🡹 | 🡹 | 1 | 2 |  |  |  |  |  |  |  |  |  |  |  |  |
| C2_3031 | Cytochrome c oxidase subunit 8A, mitochondrial | 🡹 | 🡹 | 1 | 2 |  |  |  |  |  |  |  |  |  |  |  |  |
| C2_70910 | Cytosolic sulfotransferase 1 | = | 🡻 | 16 |  |  |  |  |  |  |  |  |  |  |  |  |  |
| [C2_10068](http://nutrigroup-iats.org/seabreamdb/blastResultWww.php#247) | Delta-1-pyrroline-5-carboxylate dehydrogenase, mitochondrial | = | 🡹 | 15 | 16 |  |  |  |  |  |  |  |  |  |  |  |  |
| C2_108 | DNA damage-inducible transcript 4 protein | = | 🡹 | 4 |  |  |  |  |  |  |  |  |  |  |  |  |  |
| C2_3197 | DnaJ homolog subfamily C member 11 | 🡹 | 🡹 | 9 | 10 | 11 |  |  |  |  |  |  |  |  |  |  |  |
| C2_5322 | DnaJ homolog subfamily C member 17 | 🡹 | 🡹 | 9 | 10 | 11 |  |  |  |  |  |  |  |  |  |  |  |
| C2_5055 | DnaJ homolog subfamily C member 30 | 🡹 | 🡹 | 10 | 11 |  |  |  |  |  |  |  |  |  |  |  |  |
| C2_1371 | DnaJ homolog subfamily C member 8 | = | 🡹 | 9 | 10 | 11 |  |  |  |  |  |  |  |  |  |  |  |
| C2_5763 | E3 ubiquitin-protein ligase NEDD4-like | = | 🡻 | 11 |  |  |  |  |  |  |  |  |  |  |  |  |  |
| C2_35612 | Elongation factor 1-alpha, somatic form | = | 🡹 | 16 | 17 |  |  |  |  |  |  |  |  |  |  |  |  |
| AM955547 | Ephrin-B1 | = | 🡻 | 6 |  |  |  |  |  |  |  |  |  |  |  |  |  |
| C2_48308 | Estrogen receptor | = | 🡻 | 15 |  |  |  |  |  |  |  |  |  |  |  |  |  |
| C2_406 | Eukaryotic initiation factor 4A-I | 🡹 | = | 3 | 4 | 5 |  |  |  |  |  |  |  |  |  |  |  |
| ***Clone*** | ***Gene description*** | ***Hypo*** | ***Hyper*** | ***Canonical pathways*** | | | | | | | | | | | | | |
| C2_5966 | Eukaryotic translation initiation factor 2 subunit 2 | = | 🡹 | 3 | 5 |  |  |  |  |  |  |  |  |  |  |  |  |
| C2_533 | Eukaryotic translation initiation factor 3 subunit A | 🡹 | = | 3 | 4 | 5 |  |  |  |  |  |  |  |  |  |  |  |
| C2_92 | Eukaryotic translation initiation factor 3 subunit F | 🡹 | = | 3 | 4 | 5 |  |  |  |  |  |  |  |  |  |  |  |
| C2_5501 | Eukaryotic translation initiation factor 3 subunit K | 🡹 | = | 3 | 4 | 5 |  |  |  |  |  |  |  |  |  |  |  |
| C2_372 | Eukaryotic translation initiation factor 3 subunit L | 🡹 | = | 3 | 4 | 5 |  |  |  |  |  |  |  |  |  |  |  |
| C2_2794 | Glutamate--cysteine ligase catalytic subunit | = | 🡹 | 9 | 16 |  |  |  |  |  |  |  |  |  |  |  |  |
| C2_94546 | Glutathione S-transferase A | 🡻 | = | 9 | 15 | 16 |  |  |  |  |  |  |  |  |  |  |  |
| C2_868 | Glutathione S-transferase Mu 3 | 🡻 | 🡻 | 9 | 15 | 16 |  |  |  |  |  |  |  |  |  |  |  |
| [C2_4831](http://nutrigroup-iats.org/seabreamdb/blastResultWww.php#10289) | GTP-binding protein Rheb | 🡻 | = | 4 |  |  |  |  |  |  |  |  |  |  |  |  |  |
| C2_2112 | GTPase KRas | 🡻 | 🡻 | 3 | 4 | 5 | 6 | 7 | 8 | 9 | 10 | 12 | 13 | 14 | 16 | 17 | 18 |
| C2_829 | Guanine nucleotide-binding protein G(i) subunit alpha-2 | 🡻 | 🡻 | 6 | 17 | 18 |  |  |  |  |  |  |  |  |  |  |  |
| C2_3160 | Guanine nucleotide-binding protein subunit beta-4 | 🡻 | 🡻 | 6 | 17 | 18 |  |  |  |  |  |  |  |  |  |  |  |
| C2_58 | Heat shock 70 kDa protein 14 | 🡹 | = | 10 | 11 |  |  |  |  |  |  |  |  |  |  |  |  |
| C2_4175 | Integrin beta-1 | 🡻 | = | 5 | 6 | 7 | 8 | 13 | 14 |  |  |  |  |  |  |  |  |
| C2_5987 | Methylmalonate-semialdehyde dehydrogenase [acylating], mitochondrial | 🡹 | = | 15 | 16 |  |  |  |  |  |  |  |  |  |  |  |  |
| C2_3688 | Mitogen-activated protein kinase 14A | = | 🡻 | 5 | 9 | 12 | 13 | 16 |  |  |  |  |  |  |  |  |  |
| C2_1373 | NADH dehydrogenase [ubiquinone] 1 alpha subcomplex subunit 8 | = | 🡹 | 1 | 2 |  |  |  |  |  |  |  |  |  |  |  |  |
| C2_467 | NADH dehydrogenase [ubiquinone] 1 beta subcomplex subunit 5, mitochondrial | = | 🡹 | 1 | 2 |  |  |  |  |  |  |  |  |  |  |  |  |
| [C2_1488](http://nutrigroup-iats.org/seabreamdb/blastResultWww.php#2652) | NADH-ubiquinone oxidoreductase 75 kDa subunit, mitochondrial | 🡻 | = | 1 | 2 |  |  |  |  |  |  |  |  |  |  |  |  |
| C2_3174 | Nuclear receptor coactivator 7 | 🡹 | = | 15 |  |  |  |  |  |  |  |  |  |  |  |  |  |
| C2_466 | Peroxiredoxin-1 | 🡻 | = | 9 |  |  |  |  |  |  |  |  |  |  |  |  |  |
| C2_4821 | Peroxiredoxin-5, mitochondrial | 🡻 | = | 2 |  |  |  |  |  |  |  |  |  |  |  |  |  |
| C2_14866 | Phosphatidylinositol 3-kinase catalytic subunit type 3 | 🡻 | = | 3 | 4 | 5 | 7 | 9 | 10 | 12 | 13 | 14 | 16 | 17 | 18 |  |  |
| ***Clone*** | ***Gene description*** | ***Hypo*** | ***Hyper*** | ***Canonical pathways*** | | | | | | | | | | | | | |
| C2_52053 | Proteasome activator complex subunit 2 | 🡻 | 🡻 | 11 |  |  |  |  |  |  |  |  |  |  |  |  |  |
| C2_30726 | Protein kinase C alpha type | 🡻 | = | 4 | 9 | 10 | 12 | 16 | 17 | 18 |  |  |  |  |  |  |  |
| C2_1119 | Protein phosphatase 1 regulatory subunit 3C-B | = | 🡻 | 17 |  |  |  |  |  |  |  |  |  |  |  |  |  |
| C2_7026 | Ras-related protein R-Ras2 | = | 🡻 | 3 | 4 | 5 | 6 | 7 | 8 | 9 | 12 | 13 | 14 | 16 | 17 | 18 |  |
| C2_57583 | Ras-related protein Rap-1 | = | 🡹 | 6 | 14 |  |  |  |  |  |  |  |  |  |  |  |  |
| C2_75533 | RB1-inducible coiled-coil protein 1 | = | 🡻 | 17 |  |  |  |  |  |  |  |  |  |  |  |  |  |
| C2_2184 | Retinoic acid receptor RXR-beta-A | = | 🡻 | 15 |  |  |  |  |  |  |  |  |  |  |  |  |  |
| C2_8036 | Serine/threonine-protein phosphatase 2A 55 kDa regulatory subunit B alpha isoform | 🡹 | = | 4 | 5 | 16 | 17 |  |  |  |  |  |  |  |  |  |  |
| C2_628_u | Succinate dehydrogenase [ubiquinone] cytochrome b small subunit B, mitochondrial (KC217618) | 🡹 | 🡹 | 1 | 2 |  |  |  |  |  |  |  |  |  |  |  |  |
| C2_1571 | Succinate dehydrogenase [ubiquinone] flavoprotein subunit, mitochondrial | = | 🡹 | 1 | 2 |  |  |  |  |  |  |  |  |  |  |  |  |
| C2_1237 | Succinate dehydrogenase [ubiquinone] iron-sulfur subunit, mitochondrial | = | 🡹 | 1 | 2 |  |  |  |  |  |  |  |  |  |  |  |  |
| C2_1691 | T-complex protein 1 subunit eta | 🡹 | = | 9 |  |  |  |  |  |  |  |  |  |  |  |  |  |
| C2_13490 | Tetraspanin-5 | = | 🡻 | 14 |  |  |  |  |  |  |  |  |  |  |  |  |  |
| C2_4032 | Transcription factor p65 | = | 🡻 | 7 | 12 | 15 | 16 | 18 |  |  |  |  |  |  |  |  |  |
| C2_38179 | Tubulin alpha-1A chain | 🡻 | = | 17 |  |  |  |  |  |  |  |  |  |  |  |  |  |
| C2_17916 | Tyrosine-protein phosphatase non-receptor type 12 | 🡻 | 🡻 | 13 |  |  |  |  |  |  |  |  |  |  |  |  |  |
| C2_17030_u | Ubiquitin-conjugating enzyme E2 N (KM522797) | 🡹 | 🡹 | 11 |  |  |  |  |  |  |  |  |  |  |  |  |  |
| C2_1249 | Ubiquitin-conjugating enzyme E2 variant 1 | = | 🡹 | 11 |  |  |  |  |  |  |  |  |  |  |  |  |  |
| C2_29802 | Ubiquitin-like protein FUBI | 🡹 | = | 3 | 4 | 5 |  |  |  |  |  |  |  |  |  |  |  |
| C2_9594 | Vinculin | = | 🡹 | 13 | 14 |  |  |  |  |  |  |  |  |  |  |  |  |
| C2_1494 | Voltage-dependent anion-selective channel protein 2 | = | 🡹 | 2 |  |  |  |  |  |  |  |  |  |  |  |  |  |

**S1 File Table C. Differentially expressed genes mapped in overlapping pathway charts of gills after hypo- (Hypo) and hyper- (Hyper) challenge referred to SW acclimated fish (control, 38 ppt).** For further details, see the legend of Table 2 in S1 File.

| ***Clone*** | ***Gene description*** | ***Hypo*** | ***Hyper*** | ***Canonical pathways*** | | | | | | | | | | | | | |
| --- | --- | --- | --- | --- | --- | --- | --- | --- | --- | --- | --- | --- | --- | --- | --- | --- | --- |
| C2_440 | Alpha-enolase | = | 🡹 | 19 | 20 |  |  |  |  |  |  |  |  |  |  |  |  |
| C2_2702 | Apoptosis regulator BAX | 🡻 | = | 15 |  |  |  |  |  |  |  |  |  |  |  |  |  |
| C2_13009 | ATP synthase-coupling factor 6, mitochondrial | 🡻 | = | 1 | 2 |  |  |  |  |  |  |  |  |  |  |  |  |
| C2_1152 | Cytochrome b-c1 complex subunit 1, mitochondrial | = | 🡹 | 1 | 2 |  |  |  |  |  |  |  |  |  |  |  |  |
| C2_12715 | Cytochrome b5 | 🡹 | = | 1 | 2 |  |  |  |  |  |  |  |  |  |  |  |  |
| C2_745 | Cytochrome c oxidase subunit 5A, mitochondrial | = | 🡹 | 1 | 2 |  |  |  |  |  |  |  |  |  |  |  |  |
| C2_203_u | Cytochrome c oxidase subunit 8B, mitochondrial (KC217648) | = | 🡹 | 1 | 2 |  |  |  |  |  |  |  |  |  |  |  |  |
| C2_1367 | Cytochrome P450 1A1 | 🡹 | = | 15 | 16 | 25 | 26 | 27 | 28 |  |  |  |  |  |  |  |  |
| C2_2523 | Cytochrome P450 1B1 | = | 🡹 | 15 | 16 | 25 | 26 | 27 | 28 |  |  |  |  |  |  |  |  |
| C2_528 | Cytosolic sulfotransferase 2 | 🡹 | = | 16 | 24 | 25 | 28 | 29 |  |  |  |  |  |  |  |  |  |
| C2_1285 | Fructose-bisphosphate aldolase C-B | = | 🡹 | 19 | 20 |  |  |  |  |  |  |  |  |  |  |  |  |
| C2_2794 | Glutamate--cysteine ligase catalytic subunit | 🡹 | = | 16 |  |  |  |  |  |  |  |  |  |  |  |  |  |
| C2_117130 | Glutathione S-transferase A | 🡹 | 🡹 | 15 | 16 |  |  |  |  |  |  |  |  |  |  |  |  |
| C2_29 | Glyceraldehyde 3-phosphate dehydrogenase, testis-specific | = | 🡹 | 19 | 20 |  |  |  |  |  |  |  |  |  |  |  |  |
| C2_50666 | Heat shock protein HSP 90-alpha | 🡹 | = | 15 | 16 |  |  |  |  |  |  |  |  |  |  |  |  |
| C2_11452 | Malate dehydrogenase, cytoplasmic | = | 🡹 | 20 |  |  |  |  |  |  |  |  |  |  |  |  |  |
| C2_357 | NADH-cytochrome b5 reductase | = | 🡹 | 2 |  |  |  |  |  |  |  |  |  |  |  |  |  |
| C2_1163 | Putative adenosylhomocysteinase 3 | = | 🡹 | 21 | 22 | 23 |  |  |  |  |  |  |  |  |  |  |  |
| C2_23470 | Putative ribosomal RNA methyltransferase 1 | 🡻 | = | 21 | 22 | 23 |  |  |  |  |  |  |  |  |  |  |  |
| C2_972 | S-adenosylmethionine synthase isoform type-2 | 🡹 | = | 21 | 22 | 23 |  |  |  |  |  |  |  |  |  |  |  |
| C2_1571 | Succinate dehydrogenase [ubiquinone] flavoprotein subunit, mitochondrial | = | 🡹 | 1 | 2 |  |  |  |  |  |  |  |  |  |  |  |  |
| C2_3599 | UDP-glucuronosyltransferase (Fragment) | 🡹 | = | 16 | 24 | 25 | 26 | 27 | 28 | 29 |  |  |  |  |  |  |  |
| C2_110476 | UDP-glucuronosyltransferase 2A3 | = | 🡹 | 24 | 25 | 26 | 27 | 28 | 29 |  |  |  |  |  |  |  |  |
| C2_744 | Voltage-dependent anion-selective channel protein 2 | 🡹 | = | 2 |  |  |  |  |  |  |  |  |  |  |  |  |  |

**S1 File Table D. Differentially expressed genes mapped in overlapping pathway charts of hypothalamus after hypo- (Hypo) and hyper- (Hyper) challenge referred to SW acclimated fish (control, 38 ppt).** For further details, see the legend of Table 2 in S1 File.

| ***Clone*** | ***Gene description*** | ***Hypo*** | ***Hyper*** | ***Canonical pathways*** | | | | | | | | | | | | | |
| --- | --- | --- | --- | --- | --- | --- | --- | --- | --- | --- | --- | --- | --- | --- | --- | --- | --- |
| C2_119067 | Actin | 🡹 | = | 14 | 30 | 31 | 32 | 33 | 34 | 35 | 38 |  |  |  |  |  |  |
| C2_4352 | C-C chemokine receptor type 7 | 🡻 | 🡻 | 37 |  |  |  |  |  |  |  |  |  |  |  |  |  |
| C2_2045_u | Cadherin-1 (KF861995) | = | 🡻 | 30 | 31 | 33 | 35 |  |  |  |  |  |  |  |  |  |  |
| C2_2905_u | Calpain 3 (KM522785) | = | 🡻 | 14 | 38 | 39 |  |  |  |  |  |  |  |  |  |  |  |
| [C2_7535](http://nutrigroup-iats.org/seabreamdb/blastResultWww.php#14377) | Calpain small subunit 1 | 🡹 | = | 14 | 38 | 39 |  |  |  |  |  |  |  |  |  |  |  |
| C2_32418 | Carboxylesterase 5A | 🡹 | = | 14 |  |  |  |  |  |  |  |  |  |  |  |  |  |
| C2_2155 | Claudin-10 | 🡻 | = | 32 | 33 |  |  |  |  |  |  |  |  |  |  |  |  |
| C2_389 | Collagen alpha-1(I) chain | 🡹 | = | 36 | 37 |  |  |  |  |  |  |  |  |  |  |  |  |
| C2_17337 | Collagen alpha-1(VIII) chain | 🡹 | = | 37 |  |  |  |  |  |  |  |  |  |  |  |  |  |
| C2_70961 | Collagen alpha-1(XI) chain | 🡹 | = | 37 |  |  |  |  |  |  |  |  |  |  |  |  |  |
| C2_654 | Collagen alpha-2(I) chain | 🡹 | = | 36 | 37 |  |  |  |  |  |  |  |  |  |  |  |  |
| C2_5975 | Collagen alpha-2(V) chain | 🡹 | = | 37 |  |  |  |  |  |  |  |  |  |  |  |  |  |
| C2_5084 | Collagen alpha-3(VI) chain | 🡹 | = | 37 |  |  |  |  |  |  |  |  |  |  |  |  |  |
| C2_2523 | Cytochrome P450 1B1 | 🡹 | = | 16 | 25 | 26 | 27 | 28 |  |  |  |  |  |  |  |  |  |
| C2_4087 | Cytochrome P450 2J2 | 🡹 | = | 25 | 26 | 27 | 28 |  |  |  |  |  |  |  |  |  |  |
| [C2_528](http://nutrigroup-iats.org/seabreamdb/blastResultWww.php#11081) | Cytosolic sulfotransferase 2 | 🡹 | = | 16 | 25 | 28 |  |  |  |  |  |  |  |  |  |  |  |
| [C2_3681_u](http://nutrigroup-iats.org/seabreamdb/blastResultWww.php#17779) | Desmin (KM522780) | 🡻 | 🡻 | 35 |  |  |  |  |  |  |  |  |  |  |  |  |  |
| [C2_9271](http://nutrigroup-iats.org/seabreamdb/blastResultWww.php#16414) | Dual specificity mitogen-activated protein kinase kinase 2 | 🡹 | = | 14 | 16 | 30 | 33 | 34 | 35 | 38 | 39 |  |  |  |  |  |  |
| C2_4106 | E-selectin | 🡹 | = | 36 |  |  |  |  |  |  |  |  |  |  |  |  |  |
| C2_8464 | Extracellular superoxide dismutase [Cu-Zn] | 🡹 | = | 16 |  |  |  |  |  |  |  |  |  |  |  |  |  |
| C2_117130 | Glutathione S-transferase A | = | 🡹 | 16 |  |  |  |  |  |  |  |  |  |  |  |  |  |
| [C2_9152](http://nutrigroup-iats.org/seabreamdb/blastResultWww.php#16298) | Heat-stable enterotoxin receptor | = | 🡻 | 34 |  |  |  |  |  |  |  |  |  |  |  |  |  |
| C2_21179 | Interleukin-1 receptor-like 2 | 🡹 | = | 37 |  |  |  |  |  |  |  |  |  |  |  |  |  |
| C2_1814_u | Junctional adhesion molecule A (KF861997) | = | 🡻 | 32 | 33 |  |  |  |  |  |  |  |  |  |  |  |  |
| ***Clone*** | ***Gene description*** | ***Hypo*** | ***Hyper*** | ***Canonical pathways*** | | | | | | | | | | | | | |
| [C2_20103](http://nutrigroup-iats.org/seabreamdb/blastResultWww.php#4241) | Lamin-A | 🡻 | = | 39 |  |  |  |  |  |  |  |  |  |  |  |  |  |
| C2_11336 | Lipoprotein lipase | 🡹 | = | 36 |  |  |  |  |  |  |  |  |  |  |  |  |  |
| C2_36855 | Septin-7-like | = | 🡻 | 35 |  |  |  |  |  |  |  |  |  |  |  |  |  |
| C2_6097 | Stathmin | 🡹 | = | 35 |  |  |  |  |  |  |  |  |  |  |  |  |  |
| C2_108778 | Tetraspanin-4 | 🡹 | = | 14 |  |  |  |  |  |  |  |  |  |  |  |  |  |
| C2_2406 | Transforming growth factor beta-1 | = | 🡻 | 30 | 32 | 36 | 37 |  |  |  |  |  |  |  |  |  |  |
| C2_7711 | Tubulin alpha-1B chain | 🡻 | = | 30 | 31 | 33 | 34 |  |  |  |  |  |  |  |  |  |  |
| [C2_12213](http://nutrigroup-iats.org/seabreamdb/blastResultWww.php#1713) | UDP-glucuronosyltransferase 1-9 | = | 🡻 | 16 | 25 | 26 | 27 | 28 |  |  |  |  |  |  |  |  |  |
